# Supplementary material for: Behavioral consequences of second-person pronouns in written communications between authors and reviewers of scientific papers
Source: Nat Commun. 2024 Jan 2;15:152. doi: 10.1038/s41467-023-44515-1 (PMC10762116; doi:10.1038/s41467-023-44515-1)
Supplement: Supplementary file 1 — Supplementary Information [file 41467_2023_44515_MOESM1_ESM.pdf]

Supplementary Information

Behavioral consequences of second-person pronouns in written communications between authors and reviewers of scientific papers

Authors: Zhuanlan Sun[1,4], C. Clark Cao[2,4], Sheng Liu[2,4], Yiwei Li[2*], Chao Ma[3*]

Affiliations:

[1] High-Quality Development Evaluation Institute, Nanjing University of Posts and Telecommunications, Nanjing, China.

[2] Department of Marketing and International Business, Lingnan University, Hong Kong, China.

[3] School of Economics and Management, Southeast University, Nanjing, China.

[4] These authors contributed equally: Zhuanlan Sun, C. Clark Cao, Sheng Liu.

[*] Correspondence: victor.li@ln.edu.hk; machao@seu.edu.cn

Table of Contents

Supplementary Notes 4
Supplementary Note 1. Number of papers and rounds of review 4
Supplementary Note 2. Analysis of integrating the cascading approach with the DID model 4
Supplementary Note 3. Description of four control variables 4
Supplementary Note 4. Robustness checks 5
Supplementary Note 5. Description of two indicators of engaging conversations 6
Supplementary Note 6. Analysis of reviewers' usage of first-person pronouns 7
Supplementary Note 7. LDA topic modelling 7
Supplementary Note 8. Dictionaries for the measurements of three variables 8
Supplementary Note 9. Split the DDD analysis into two respective DIDs 9
Supplementary Methods 10
Supplementary Method 1. Post hoc gender-based analysis of the behavioral experiment (main text) 10
Supplementary Method 2. Behavioral experiment (replication) 10
Supplementary Figures 12
Supplementary Figure 1. Distribution of number of words and number of questions 12
Supplementary Figure 2. A cascade view of the mean word count (WC) and mean question count (QC) in the review process 13
Supplementary Figure 3. Kernel density maps of PSM matching 14
Supplementary Figure 4. Placebo test of four sentiments of reviewers' comments 15
Supplementary Figure 5. Word cloud of subjective words used by the reviewers 16
Supplementary Figure 6. Perplexity scores for LDA models with different number of topics 17
Supplementary Figure 7. The 10 highest probability words in each of the latent 40 topics 18
Supplementary Figure 8. Distribution of document-level topic proportions within the chosen reviewer engagement topic 19
Supplementary Figure 9. Distribution of model residuals and their variances 20
Supplementary Figure 10. Results of replication experiment 21
Supplementary Tables 22
Supplementary Table 1. Total number of papers in each round of review 22
Supplementary Table 2. Detailed description and summary statistics of four control variables 23
Supplementary Table 3. DID estimates with continuous usage of "you" 25
Supplementary Table 4. DID estimates with treatment group excluding courteous usage of "you" 26
Supplementary Table 5. DID estimates with the usage of "the reviewer" as control group 27
Supplementary Table 6. Mean differences before and after matching 28
Supplementary Table 7. Estimation results with PSM-DID approach 31
Supplementary Table 8. Estimation results with Heckman model (first stage) 32
Supplementary Table 9. Estimation results with Heckman model (second stage) 32

Supplementary Table 10. Qualitative evidence of some examples with more and less subjective sense 33
Supplementary Table 11. Qualitative evidence of some examples with complex and simple words 34
Supplementary Table 12. Different use of singular and plural first-person pronouns by reviewers 35
Supplementary Table 13. Estimation results with alternative number of topics 36
Supplementary Table 14. Estimation results with alternative topics in the latent 40 topics 37
Supplementary Table 15. Vocabulary for calculating the frequency of negative words, "high-engagement" words, and friendly words used by authors in 1st review round 38
Supplementary Table 16. Estimates for a "you" conversation initiated by reviewers 40
Supplementary Table 17. Estimates for a "you" conversation not initiated by reviewers .. 41
Supplementary Table 18. Estimates for a "you" conversation initiated by reviewers on six behavioral outcomes 42
Supplementary Table 19. Estimates for a "you" conversation not initiated by reviewers on six behavioral outcomes 43
Supplementary References 44

Supplementary Notes

Supplementary Note 1. Number of papers and rounds of review

Additional notes on number of papers and rounds:

1. As explained in the Data and Design subsection under the Results section, our initial, full data set includes 13,359 papers. These 13,359 papers account for a total of 29,144 rounds of review, defined as one exchange between the reviewers and the authors, with the reviewer comments being followed by the author responses.
2. All 13,359 papers enter our DID model. Specifically:
   a. For the 12,320 papers (see Supplementary Table 1) that underwent *at least two rounds* of review, the DID model includes their reviewer comments from the 1st and 2nd rounds.
   b. For the 1,039 papers that were accepted after *only one round* of review, the DID model includes only the reviewer comments from the 1st round (because the 2nd round reviewer comments do not exist).
3. Our DID model thus begins with a total of 25,679 observations of reviewer comments (12,320 papers × 2 rounds per paper + 1,039 papers × 1 round per paper). Our "basic" DID estimations (i.e., without fixed effects and other controls) are conducted based on this sample size (i.e., Columns (1) and (3) in Table 2 of the main text).
4. When paper fixed effects are included, the DID model further drops singleton observations that arise from these 1,039 accepted papers, leading to a final sample size of 24,640 observations (25,679 - 1,039). All remaining DID estimations reported in the manuscript are based on these 24,640 observations.

Supplementary Note 2. Analysis of integrating the cascading approach with the DID model

To provide a fuller picture of our data, we demonstrate how two of our key variables, word count and question count, bifurcate following "you" usage and cascade down as the peer review progresses. Supplementary Fig. 2 depicts the chained consequences of the "you" usage.

Supplementary Note 3. Description of four control variables

In our Difference-in-Differences (DID) model (Equation 2 in the "Methods" section), we controlled for a variety of control variables, as described in Table 1. Among these controls, four are sets of dummy variables four variables: *last initial of the first author*, *month of publication*, *publication year*, and *paper discipline*. Details of these dummy variables are summarized in Supplementary Table 2.

Supplementary Note 4. Robustness checks

We have conducted the following six robustness checks to further buttress the robustness of our findings. Their details are reported below.

Robustness Check 1: More "you" usage is associated with stronger effect. Robustness check 1 examines whether the frequency of "you" usage influences "you" usage's effectiveness. To do this, we categorize responses into groups based on the frequency of "you": a few (one or two), moderate (three through five), and many (six or more) "you" usage. We also designate responses without any "you" as our reference group. We then re-estimated the benchmark model and report the results in Supplementary Table 3. In a nutshell, the effects of "you" usage on nearly all outcomes amplify as the frequency of "you" rises, save for reviewers' positivity captured by the Python package TextBlob (but not by the R package sentimentr).

Robustness Check 2: Excluding courteous usage of "you." In the main regression (Table 3), a paper is categorized into the treatment group so long as the word "you" presents in the 1st round author response, regardless of the context or circumstance in which "you" is applied. The treatment group can thus both "courteous you" (such as "Thank you") and "non-courteous you." However, since courteous "you" usage are frequently thrown around as formality (or even cliché), it may not necessarily produce a personal, engaging conversation.

Robustness check 2 addresses this possibility. Specifically, to construct a cleaner treatment group, we include a paper in the treatment group only the "usage" usage is conversational (as opposed to courteous, e.g., "thank you"). Of all 5,042 "you" papers, 1,847 samples (36.63%) only contain courteous "you." We exclude these 1,847 courteous "you" papers from our dataset and re-estimated our regression models. The results are displayed in Supplementary Table 4, echoing our main findings.

Robustness Check 3: Using third-person addresses (e.g., "the reviewer") as control group. While Robustness Check 2 attempts to build a cleaner treatment group, Robustness Check 3 aims to build a cleaner control group. In our sample, the non-"you" author responses can be further divided into two categories, wherein authors either (a) instead used third-person language (i.e., "the reviewer") to address the reviewer, or (b) did not use second- or third-person addresses – perhaps only engaged in addressing the questions. Here, we exclude category (b) from the dataset and re-estimate our models. The corresponding estimation in Supplementary Table 5 again consistent with the main findings in Table 3 in main text.

Robustness Check 4: propensity score matching (PSM) to establish comparable treatment and control groups. To alleviate the concern that authors' "you" usage may not be sufficiently random (exogenous), we utilized propensity score matching (PSM) to establish comparable treatment and control groups for model estimation. By combining the DID approach with the PSM matching techniques, we aim to obtain "matched" experiment and control groups, which possess comparable observable characteristics. Most covariates imbalance between the two groups (Supplementary Table 6) are no longer statistically significant after matching, indicating that the 1:1 nearest neighbor PSM matching algorithm efficiently reduce the bias associated with the observable characteristics. More details on the kernel density maps of PSM before and after matching are provided in Supplementary Fig. 3. Again, consistent results are obtained through the use of the PSM-DID approach. (Supplementary Table 7).

Robustness Check 5: Heckman model. Because authors may use "you" in response to the initial use of "you" by reviewers, one may be concerned that our estimation suffers from potential self-selection bias. Robustness Check 5 implements a Heckman two-stage model to alleviate this concern[1,2]. Specifically, the first-stage model predicts authors' "you" usage, based on covariates such as author features including gender and rank, and the reviewers' "you" usage in the 1st round (i.e., "initial reviews").

For gender determination, we probabilistically inferred the gender of authors from their names, utilizing the Social Security Administration (SSA) database[3–5]. As for rank, as it is impractical to manually retrieve precise professorship details of more than ten thousand authors in our dataset, we approximate author rank using the H-index (a citation-centric metric denoting scientific impact) supplemented from the Web of Science database. This process generates the inverse Mills ratio (IMR) representing unobserved determinants of authors' "you" usage.

The second-stage main model then formally estimates the effects of authors' "you" usage, correcting for the self-selection by controlling for the IMR from the first stage. The resulting estimated effects remain consistent and robust, offering further confidence in our findings (Supplementary Table 8 and Supplementary Table 9).

Robustness Check 6: Placebo test. To further enhance the credibility of our results, we conducted a placebo test. Specifically, we assigned Response with "You" to our observations in a random manner (referred to as "placebo "you" usage": wherein the treatment group is randomly generated, allowing replacement and with group size unchanged as 38% of the papers). Subsequently, we replicated the baseline DID regression using the generated "pseudo-you" data, obtaining estimates for the beta coefficient of the key variables (interaction term in Equation 2). This process was repeated 500 times, yielding a corresponding distribution of 500 beta coefficient estimates, which is illustrated in Supplementary Fig. 4 (where the vertical dashed line in the figure corresponds to the coefficient from the Table 3).

As expected, since the placebo "you" usage is generated randomly, the expected value of the beta coefficient estimates should be close to zero. Supplementary Fig. 4 reveals that the distribution of the estimates (of the placebo "you" usage) is centered around zero, and as expected, our benchmark estimates clearly lie outside the range of the placebo estimates. This further bolsters our confidence in our DID findings, such that these findings are not driven by other unobservable factors.

Supplementary Note 5. Description of two indicators of engaging conversations

Subjectivity. Following the definition by Bravo[6], "subjectivity" captures the extent to which a text contains personal opinions rather than factual information. In this study, the subjectivity of each peer review report was obtained using the TextBlob python package. The TextBlob package employs a built-in lexicon to determine how subjective a text is on a scale ranging from 0 (very objective) to 1.0 (very subjective). Higher subjectivity scores indicate that the text is more opinionated and subjective in nature, whereas lower subjectivity scores suggest that the text is predominantly objective without much personal opinions.

To provide readers with a sense of what linguistic markers are deemed as "subjective" by TextBlob in our dataset, here we present the top 100 subjective marker words identified in reviewer comments (Supplementary Fig. 5). Notably, among these words, "different," "important," "interesting," "clear," and "new" are the top 5 subjective markers found in our reviewer comments data, highlighted in red.

The word cloud above lists key subjective markers and their relative weights in our data. However, these words are also presented in an isolated, decontextualized fashion. To this end, in Supplementary Table 10, we hand-picked a few sentences from our reviewer comments data, and calculated the subjectivity scores for those sentences using TextBlob. We believe that this *in situ* presentation renders the subjective score more sensible and relatable. Note that these example sentences are for illustrative purposes only, as our data analyses calculated subjective scores based on the full reviewer comments instead of a single sentence.

Word Complexity. Intuitively, words with more syllables also tend to be more complicated and difficult to understand. In our analysis, we measured a reviewer comment's word complexity by calculating the average number of syllables per word in that comment.

In our dataset, the mean Word Complexity value of the reviewer comments is 1.954 syllables (SD = 0.133). As with subjectivity scores, in Supplementary Table 11 we showcase examples of complex and simple sentences from our reviewer comment data, accompanied by their respective complex scores. Again, please note that our actual analyses calculated word complexity on a reviewer comment (instead of sentence level).

Supplementary Note 6. Analysis of reviewers' usage of first-person pronouns

Here we apply our DID models on reviewers' usage of first-person pronouns, both plural and singular. The results in Supplementary Table 12 suggest, when reviewers used significantly less singular first-person pronouns (indicating less egocentrism or self-focus), their use of plural first-person pronouns maintained pretty much the same before and after the authors' response with "you."

Supplementary Note 7. LDA topic modelling

We employ LDA analysis in our research to shed light on the underlying mechanism (i.e., engagement) of our findings via a data-driven approach. Specifically, LDA analysis explores potential latent topics and identify a particular topic which happens to be highly associated with the engagement during the review process.

We employed the (minimum) perplexity score to determine the optimal number of topics. Specifically, after experimenting with topic counts ranging from 10 to 100 (at 10-topic intervals), we determined 40 to be the optimal number in that it has the lowest perplexity score (Supplementary Fig. 6).

For each of the 40 latent topics, we present the top 10 words based on their probability. Amongst the 40 topics, topic11 is the identified topic related to reviewer engagement (as shown in Supplementary Fig. 7). The document-level topic proportions of reviewer engagement topic are presented in Supplementary Fig. 8. The

low prevalence of the engagement topic (1.1%) is to be expected: The low prevalence of the engagement topic (1.1%) is to be expected: After all, there are as many as 40 topics exist in the text; Moreover, the majority of said text is more likely to be languages oriented towards substantial matters of the research than those employed to engage with people. On the other hand, the distribution reveals significant diversity in the topic, as evidenced by a standard deviation of 2.5% and a maximum value of 41.9%. These variabilities provide ample opportunities for discerning the impact of the "you" usage.

Several robustness checks for LDA. Considering the potential impact of topic count (n = 40 in our study) on LDA results, we conducted the first robustness test for our LDA models with varying the number of topics (35 and 45 topics, respectively; Columns (1) and (2) in Supplementary Table 13), and the results remained robust.

Second, in addition to employing the LDA to uncover latent topics within the peer review reports, we manually compiled a list of "high-engagement" words (e.g., "exciting," "interesting," and "enjoy"; a total of 116 words listed in Supplementary Table 15) based on word counts. We used this new set to reassess the impact of the "you" usage on engagement. The results once again support a positive association between the "you" usage and engagement (Columns (3) in Supplementary Table 13), albeit with slightly reduced significance. We suspect that this diminished significance might stem from the challenge in formulating a predetermined (as opposed to data-driven) word list that effectively captures spontaneous, real-world engagement. For example, while a predetermined word list might use descriptive terms like "engage" or "collaborate," organic conversations often involve context-specific phrases like "your revision addressed my concern" or "following your recommendations."

Third, the topic model is estimated using both the treatment and control groups. It is possible that the control group may have a greater impact on the determination of topics. Therefore, we have alternatively constructed a structural topic model (STM) which considers the source of a review comment (treatment or control group) as a factor in the model's estimation. As is shown in Columns (4) in Supplementary Table 13, although the estimated effect is not as substantial (p = 0.168) as in the original LDA model, the direction of the effect remains the same, which overall aligns with proposed account.

Lastly, we have also tested our models with the engagement topic (topic 11) replaced by unrelated topics. These unrelated topics, which are used as "placebos," included topics 13 and 26, which pertain to specific scientific fields (presumably electromagnetism and ecology), and topics 18 and 36, which relate to manuscript evaluation (likely in terms of exposition and methodology). The results in Supplementary Table 14 reveal no significant correlation between the use of "you" and these unrelated topics This result further strengthens our confidence in the validity of the proposed engagement mechanism.

Supplementary Note 8. Dictionaries for the measurements of three variables

Three variables, including the Negativity (Hand Coded), "High-engagement" Words, and Friendliness of Authors (1st Round) were constructed using manually created dictionaries. The 92 negative words was employed to create the variable Negativity (Hand Coded). As outlined in the methodology, this variable

involves calculating the frequency of these 92 negative words appearing in our peer review reports (normalized by dividing by 100 for scaling purposes).

The "High-engagement" Words lexicon was developed by tallying words in peer review reports that were pertinent to reviewer engagement, combined with existing engagement lexicons (including Oxford Languages, Cambridge Dictionary, Merriam-Webster Dictionary, Collins Dictionary, Thesaurus.com, and WordHippo), yielding a total of 116 terms highly relevant to reviewer engagement. In the same vein, the Friendly of Authors lexicon was also created using a comparable approach, encompassing 178 words used to calculate the frequency of friendly words used by authors in the first round of review (Supplementary Table 15).

Supplementary Note 9. Split the DDD analysis into two respective DIDs

In this paper, we explained that a DDD model can be thought of as two separate yet comparable DIDs. Following this perspective, we present the results of DDD analyses on the four mechanism measurements of this research (i.e., subjectivity, first-person singular pronouns usage, word complexity, and LDA identified engagement topic). Note we split each DDD as two DIDs by whether reviewers used "you" in 1st round reviewer comments (see Supplementary Table 16 and Supplementary Table 17).

For comprehensiveness of analysis, here we also report DDD analyses on our six behavioral outcomes, which are also split into DID pairs (see Supplementary Table 18 and Supplementary Table 19).

Supplementary Methods

Supplementary Method 1. Post hoc gender-based analysis of the behavioral experiment (main text)

While gender-based analyses were not planned for this research, here we report *post hoc* gender-disaggregated results of the analyses detailed in the main text. These additional analyses were conducted and reported in accordance with the Sex and Gender Equity in Research (SAGER) guidelines and the editorial policies of the Nature Portfolio as of November 12, 2023.

Of the 1,601 participants, 901 (56.3) self-identified as female. Within this subgroup, an ANOVA reveals that participants in the "you" condition rated the author's response more positively (M = 5.82, SD = 0.93) than those in the non-"you" condition (M = 5.65, SD = 1.00; $F(1, 899) = 7.07$, $p = 0.008$, Cohen's $d = 0.18$, 95% CI = [0.05, 0.31]). Additionally, "you" (vs. non-"you") usage also led participants to perceive their exchange with the author as more personal and engaging (M = 5.12, SD = 1.07 vs. M = 4.78, SD = 1.24; $F(1, 899) = 18.71$, $p < 0.001$, Cohen's $d = 0.29$, 95% CI = [0.16, 0.42]). A mediation analysis shows that the relationship between "you" usage and positivity is fully mediated by participants' perception of an personal and engaging conversation (unstandardized indirect effect = 0.17, SE = 0.04, 95% CI = [0.09, 0.25]; 5,000 bootstrap resamples).

Of the 1,601 participants, 676 (42.2%) self-identified as male. Within this subgroup, an ANOVA reveals participants in the "you" condition rated the author's response more positively (M = 5.71, SD = 1.04) than those in the non-"you" condition (M = 5.54, SD = 1.04; $F(1, 674) = 4.33$, $p = 0.038$, Cohen's $d = 0.16$, 95% CI = [0.01, 0.31]). Additionally, "you" (vs. non-"you") usage also led participants to perceive their exchange with the author as more personal and engaging (M = 5.17, SD = 1.11 vs. M = 4.71, SD = 1.23; $F(1, 674) = 25.87$, $p < 0.001$, Cohen's $d = 0.39$, 95% CI = [0.24, 0.54]). A mediation analysis shows that the relationship between "you" usage and positivity is fully mediated by participants' perception of an personal and engaging conversation (unstandardized indirect effect = 0.25, SE = 0.05, 95% CI = [0.15, 0.35]; 5,000 bootstrap resamples).

Of the 1,601 participants, 24 (1.5%) self-identified as non-binary or chose not to disclose their gender. In compliance with the SAGER guidelines, within this subgroup we have refrained from conducting *post hoc* gender-based analyses, as the sample size is insufficient to enable meaningful conclusions.

Supplementary Method 2. Behavioral experiment (replication)

The goal of the replication study is two-fold. First, we demonstrate the validity of our experiment via exact replication. Second, we examine whether "you" usage invoked alternative psychological process.

We recruited 1,200 Amazon Mechanical Turk panelists via the CloudResearch platform. Of all participants, 625 (52.1%) self-identified as female, 564 (47.0%) as male, and 11 (0.9%) as non-binary; M_{age} = 39.9 years.

The first parts of this replication are identical to those of the main text experiment.

An ANOVA reveals that participants in the "you" condition rated the author's response more (M = 5.80, SD = 0.98) than those without (M = 5.66, SD = 1.01; $F(1, 1198) = 5.64$, $p = 0.018$, Cohen's d = 0.14, 95% CI = [0.02, 0.25]). Supplementary Fig. 10 (a) illustrates this result.

Additionally, "you" (vs. non-"you") usage also led participants to perceive their exchange with the author as more personal and engaging (M = 5.19, SD = 1.13 vs. M = 4.80, SD = 1.28; $F(1, 1198) = 30.68$, $p < 0.001$, Cohen's d = 0.32, 95% CI = [0.21, 0.43]). Supplementary Fig. 10 (b) illustrates this result.

We also conducted a mediation analysis. The result shows that the relationship between "you" usage and positivity is fully mediated by participants' perception of an personal and engaging conversation (unstandardized indirect effect = 0.17, SE = 0.03, 95% CI = [0.11, 0.23]; 5,000 bootstrap resamples).

Taken together, these results replicate those of the main text experiment, thus bolstering our confidence in our experiments.

Additionally, to examine whether "you" usage invoked alternative psychological process, we include measurements for three variables:

(1) Contentiousness of the communication. This was measured on a 3-item, 7-point Likert scale (1 = strongly disagree; 7 = strongly agree; Cronbach's α = 0.90): "The conversation between the parties feels contentious," "The correspondence between the reviewer and the author does NOT feel very friendly," and "I find the author's responses combative." An ANOVA reveals no statistically significant difference between the "you" and non-"you" conditions (M = 2.59, SD = 1.50 vs. M = 2.67, SD = 1.40; $F(1, 1198) = 1.10$, $p = 0.295$, Cohen's d = -0.06, 95% CI = [-0.17, 0.05]).

(2) Reviewer's perceived connection with the author. This was measured on a 2-item, 7-point Likert scale (1 = strongly disagree; 7 = strongly agree; inter-item r = 0.87): "I sense a connection with the author," "I perceive a certain bond with the author." An ANOVA reveals no statistically significant difference between the "you" and non-"you" conditions (M = 4.29, SD = 1.49 vs. M = 4.24, SD = 1.44, $F(1, 1198) = 0.33$, $p = 0.568$, Cohen's d = 0.03, 95% CI = [-0.08, 0.15]).

(3) Reviewer's sense of duty towards the author. This was measured on a 2-item, 7-point Likert scale (1 = strongly disagree; 7 = strongly agree; inter-item r = 0.87): "I feel some sort of obligation towards the author," "I develop a sense of duty towards the author." An ANOVA reveals no statistically significant difference between the "you" and non-"you" conditions (M = 4.14, SD = 1.54 vs. M = 4.14, SD = 1.61, $F(1, 1198) < 0.001$, $p = 0.983$, Cohen's $d < 0.01$, 95% CI = [-0.11, 0.11]).

In a nutshell, we observe no statistically significant effect of "you" usage on contention, personal connection, or perceived duty. Including any of these three variables as a covariate does not significantly change the results of the aforementioned ANOVA or the mediation analyses.

For this replication study, following the SAGER guidelines we have refrained from conducting *post hoc* gender-based analyses, as the sample size may be insufficient to enable meaningful conclusions.

For more details of the experimental design, we also share the experiment material on the Open Science Framework repository (https://doi.org/10.17605/OSF.IO/XWYS4).

Supplementary Figures

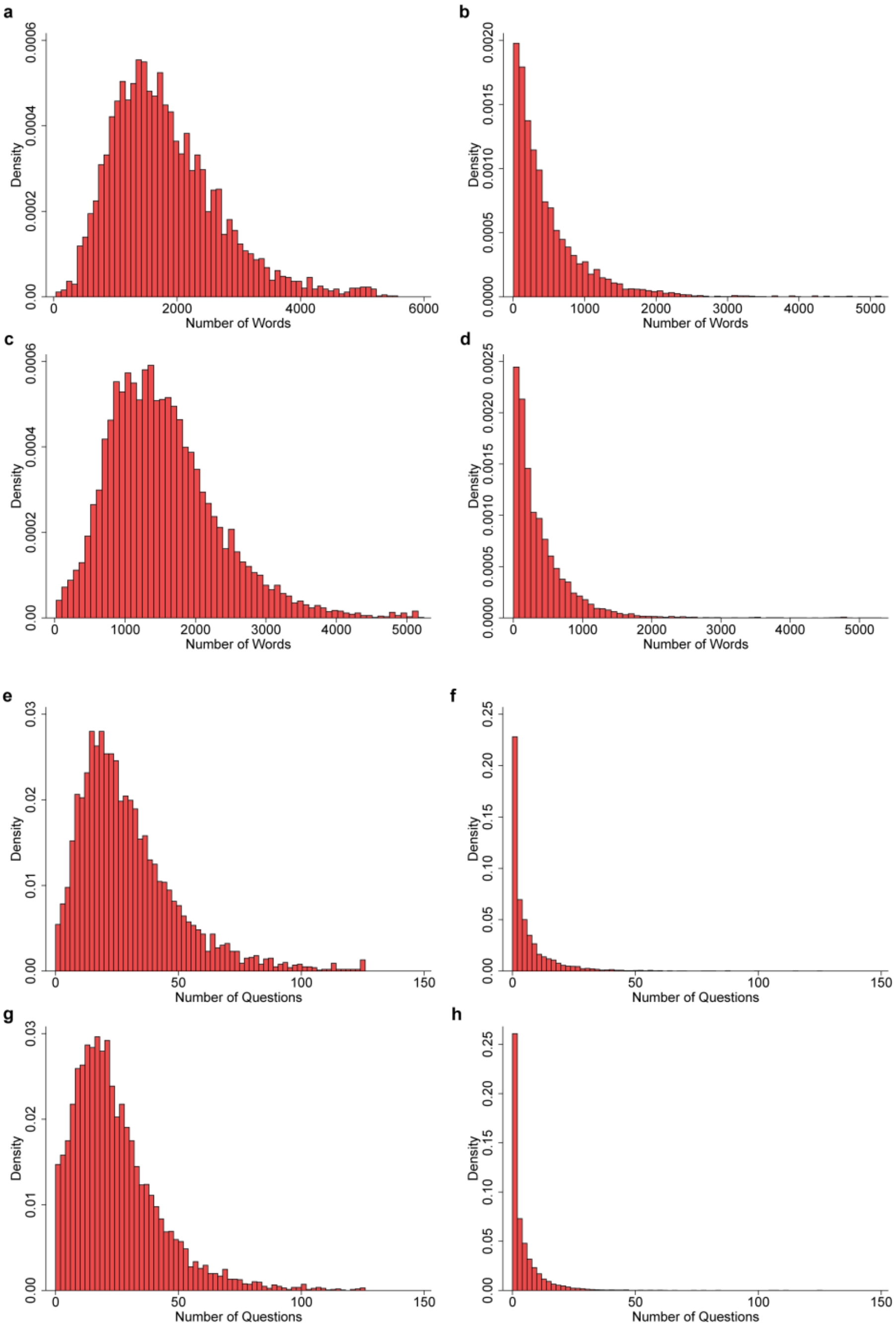

Supplementary Figure 1. Distribution of number of words and number of questions. The upper panel consists of four histograms showing the distribution of number of words in the following groups: (**a**) response with "you" and before response, (**b**) response with "you" and after response, (**c**) response without "you" and before response, and (**d**) response without "you" and after response. The lower panel comprises four histograms showing the distribution of number of questions in the following groups: (**e**) response with "you" and before response, (**f**) response with "you" and after response, (**g**) response without "you" and before response, and (**h**) response without "you" and after response. Number of questions greater than 126 only account for 0.06% of the sample, which we merge as one group.

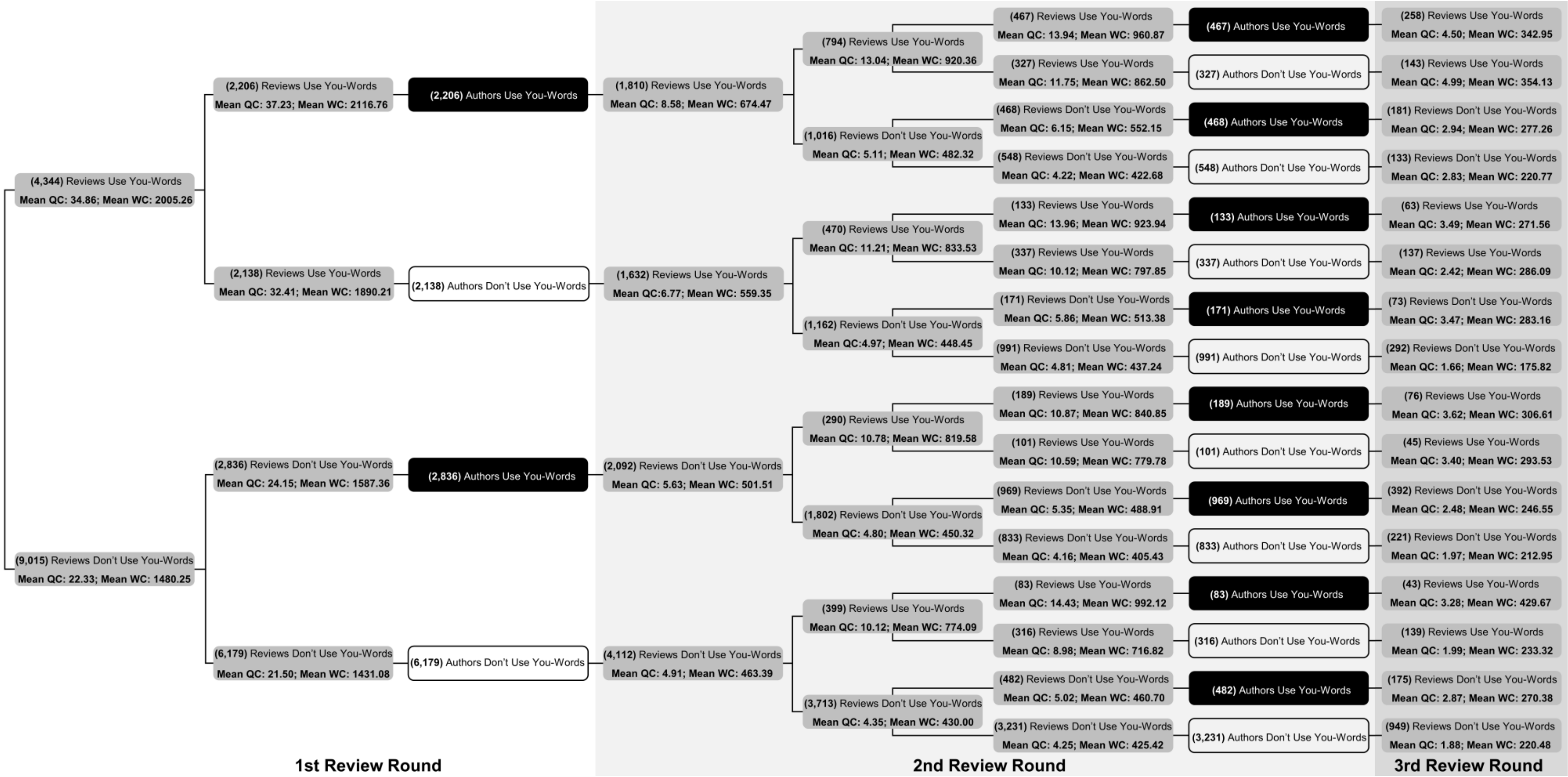

Supplementary Figure 2. A cascade view of the mean word count (WC) and mean question count (QC) in the review process. The mean WC and mean QC are reported based on whether the reviewers use you or not in the 1st, 2nd, and 3rd review round. For higher resolution, please visit https://doi.org/10.17605/OSF.IO/XWYS4.

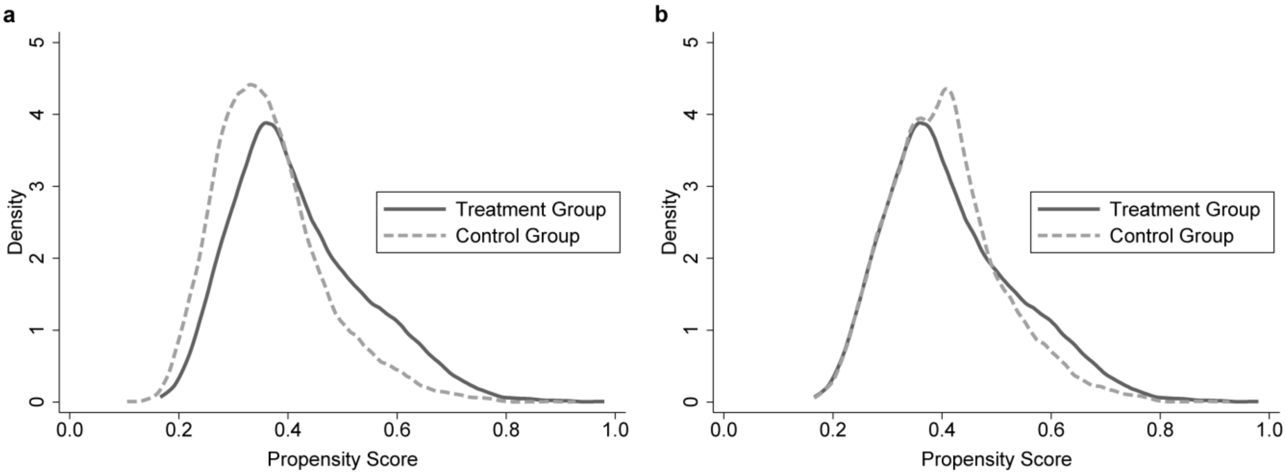

Supplementary Figure 3. Kernel density maps of PSM matching. (**a**) Kernel density maps of PSM before matching. (**b**) Kernel density maps of PSM after matching.

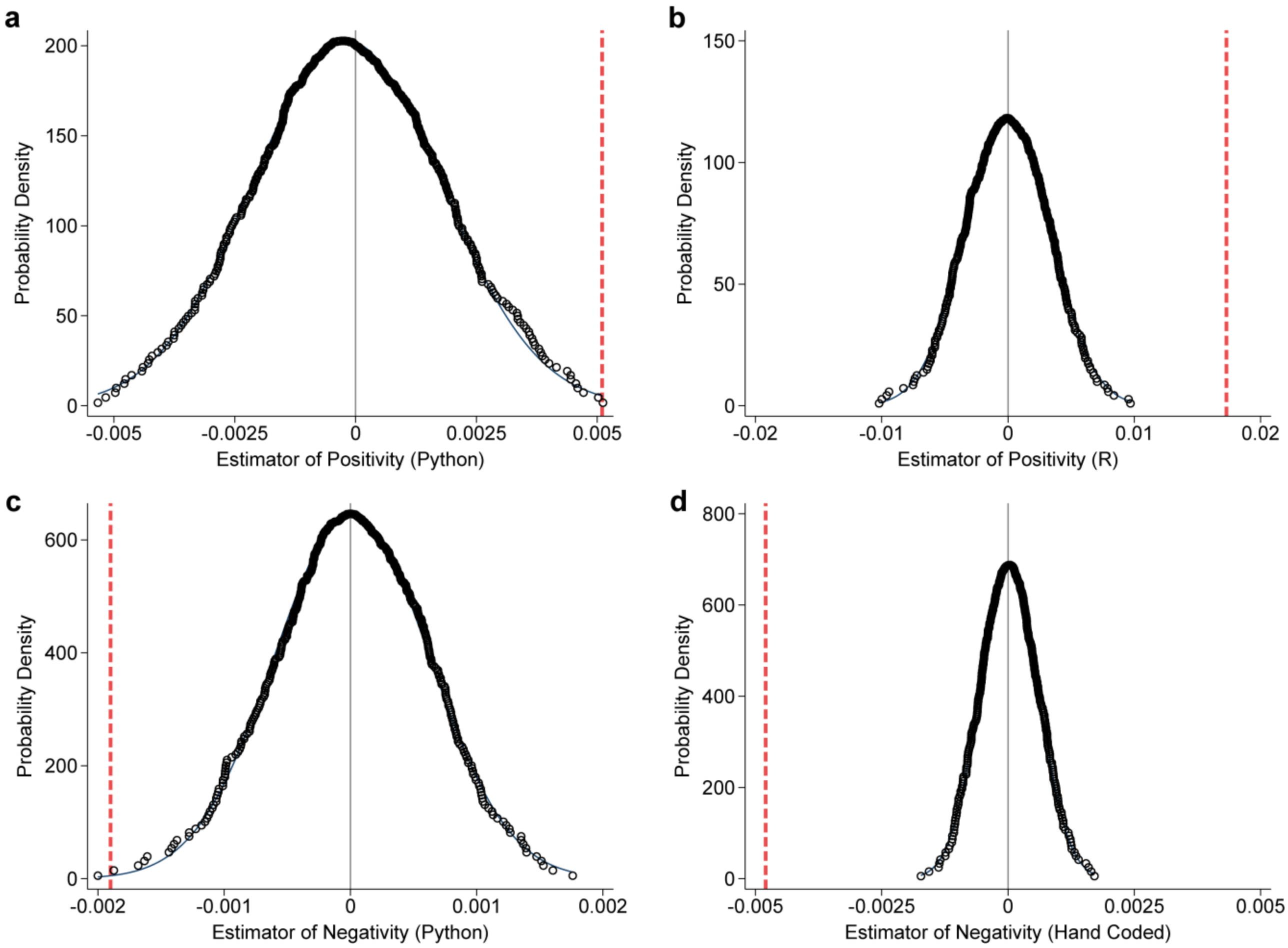

Supplementary Figure 4. Placebo test of four sentiments of reviewers' comments. The distribution of beta coefficients using a "placebo 'you' usage" repeated 500 times in the DID regression forms the four figures. Vertical dashed line in the figure corresponds to the coefficient from the Table 3. The distribution of Beta coefficients for (**a**) Positivity (Python), (**b**) Positivity (R), (**c**) Negativity (Python), and (**d**) Negativity (Hand Coded).

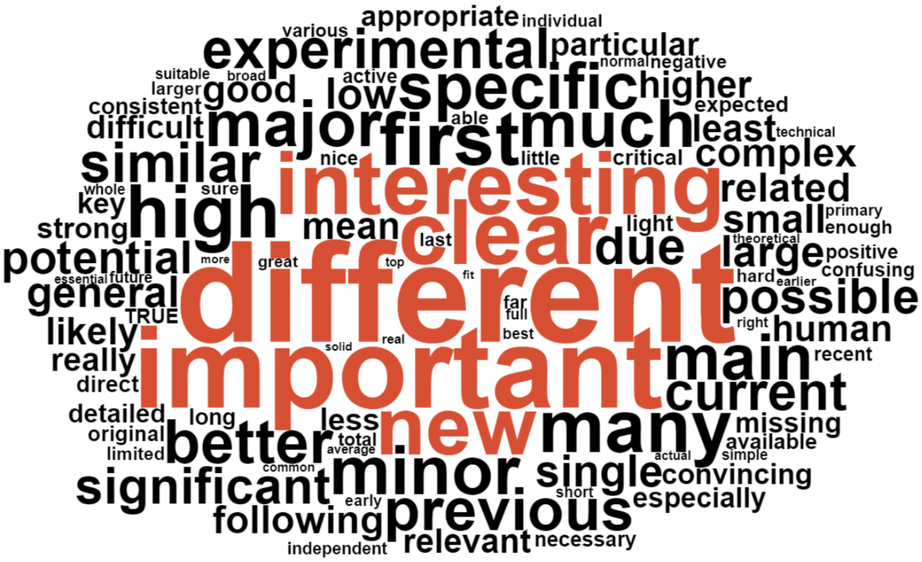

Supplementary Figure 5. **Word cloud of subjective words used by the reviewers.** The word size represents the (relative) frequency of words in the reviewer comments. The top 5 words (in red color) are “different,” “important,” “interesting,” “clear,” and “new.”

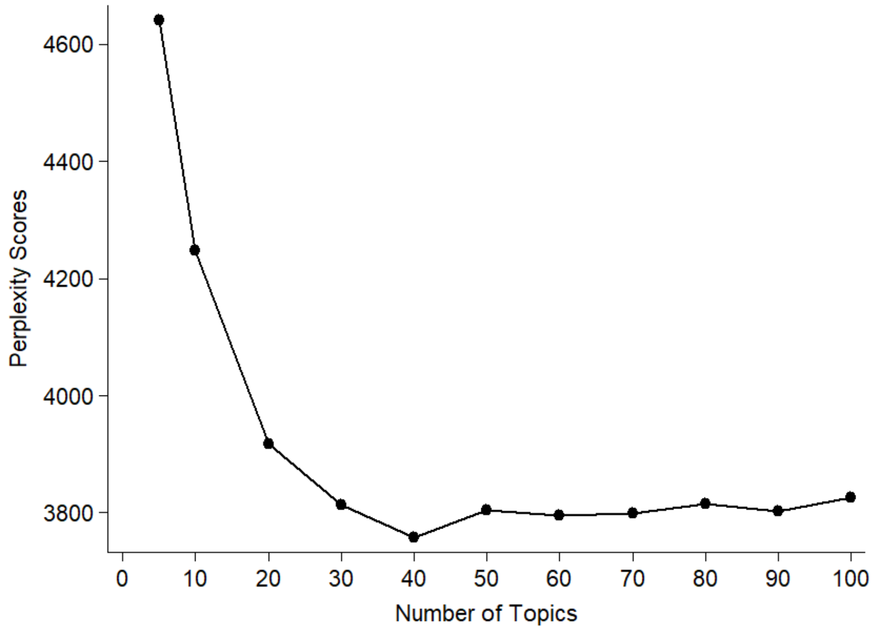

Supplementary Figure 6. Perplexity scores for LDA models with different number of topics. The generated perplexity scores suggest the optimal number of topics to be 40.

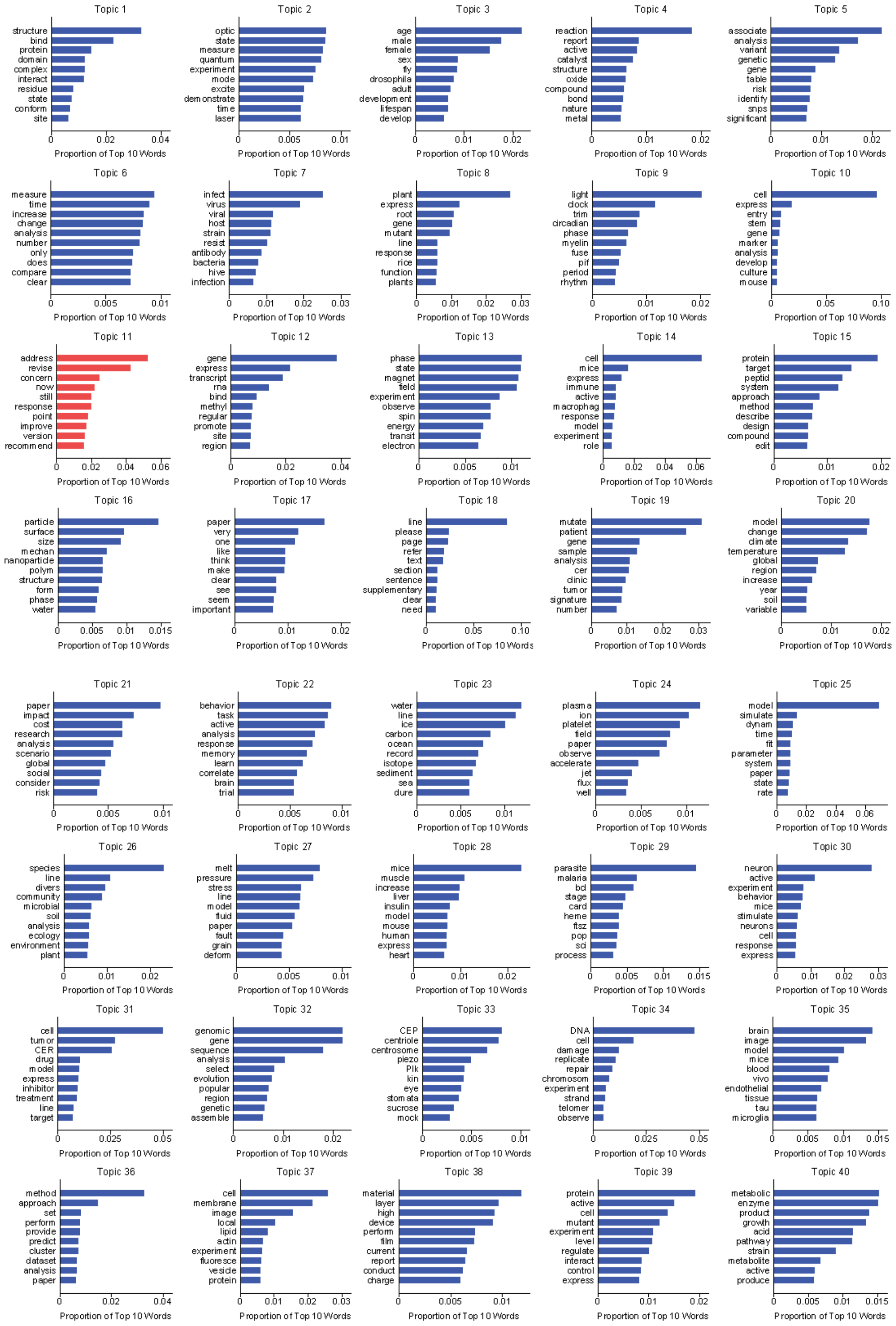

Supplementary Figure 7. The 10 highest probability words in each of the latent 40 topics. Latent Dirichlet Allocation (LDA) model generates the 40 topics and the top 10 highest probability words in each of the topics. Topic 11 with red bars is the engagement topic of our interest.

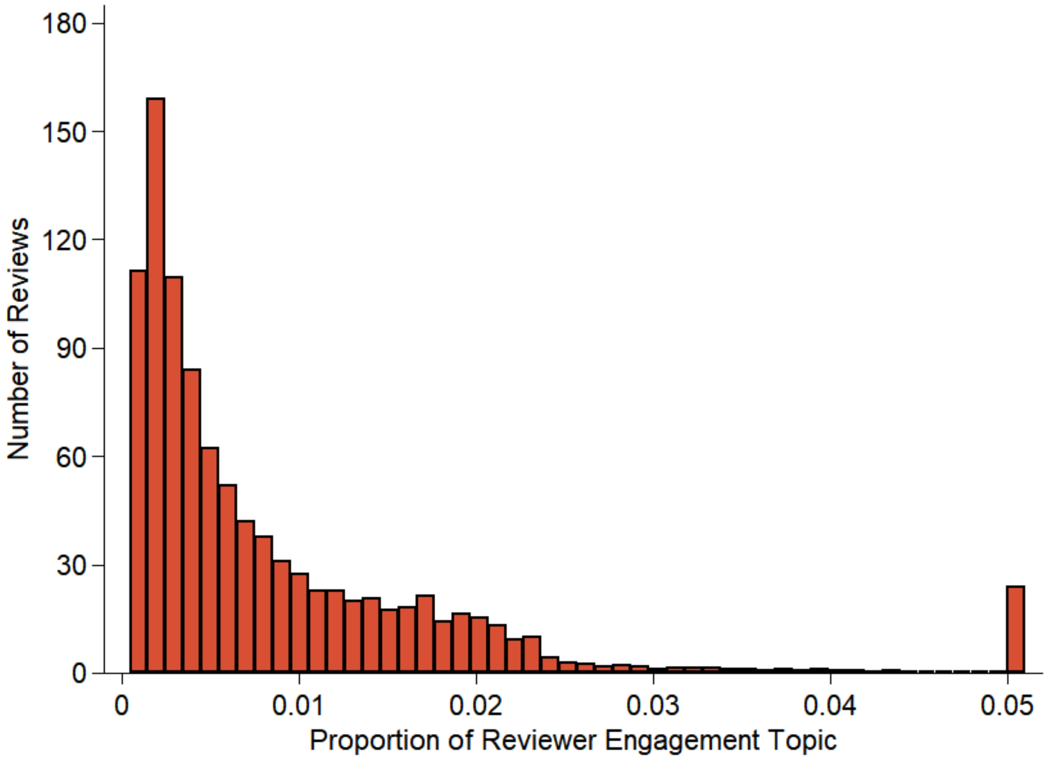

”po

Supplementary Figure 8. Distribution of document-level topic proportions within the chosen reviewer engagement topic. Document-level topic proportions greater than 0.05 only account for 2.38% of the sample, which we merge as one group.

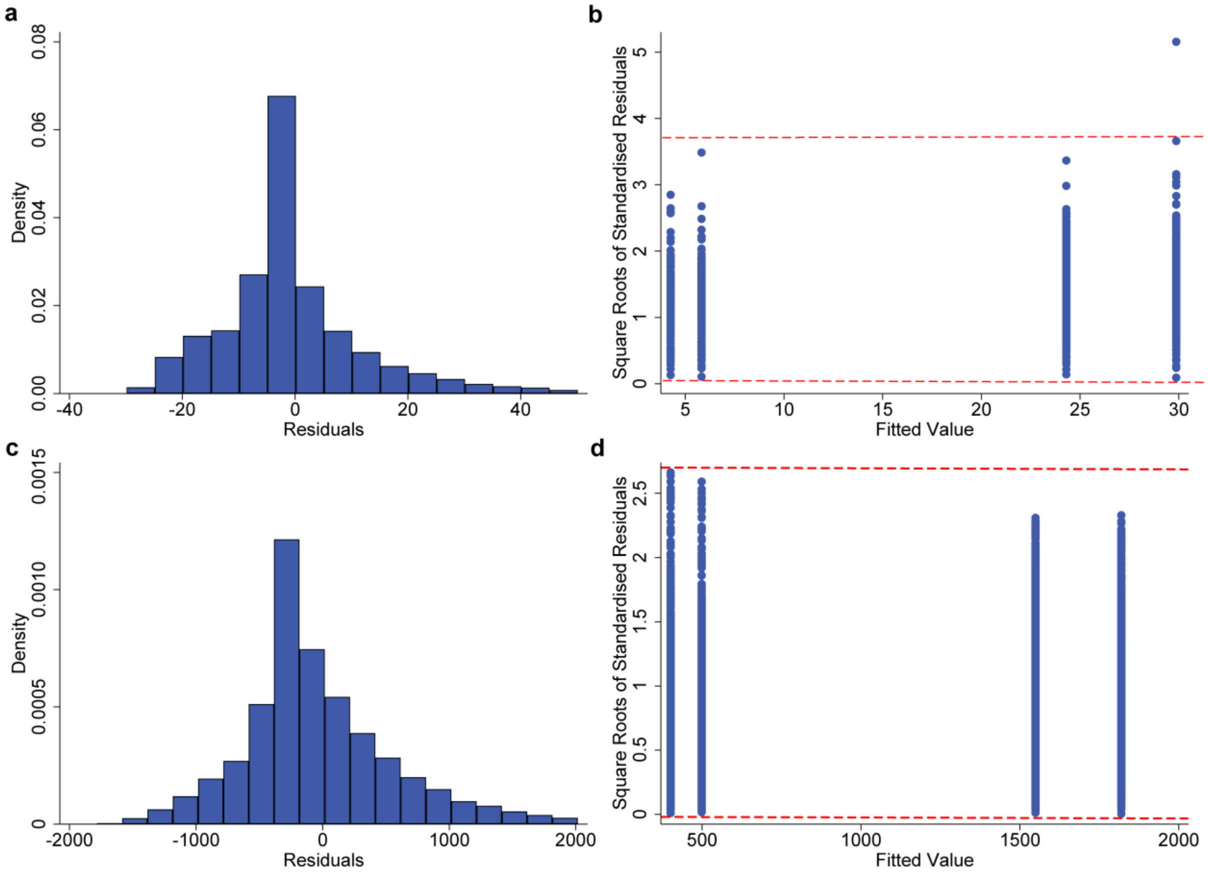

Supplementary Figure 9. Distribution of model residuals and their variances. For the dependent variable - Number of Questions (column 1 in Table 2): (**a**) histogram of the residual, (**b**) square root of standardized residuals against fitted values. For the dependent variable - Number of Words (column 3 in Table 2): (**c**) histogram of the residual, (**d**) square root of standardized residuals against fitted values.

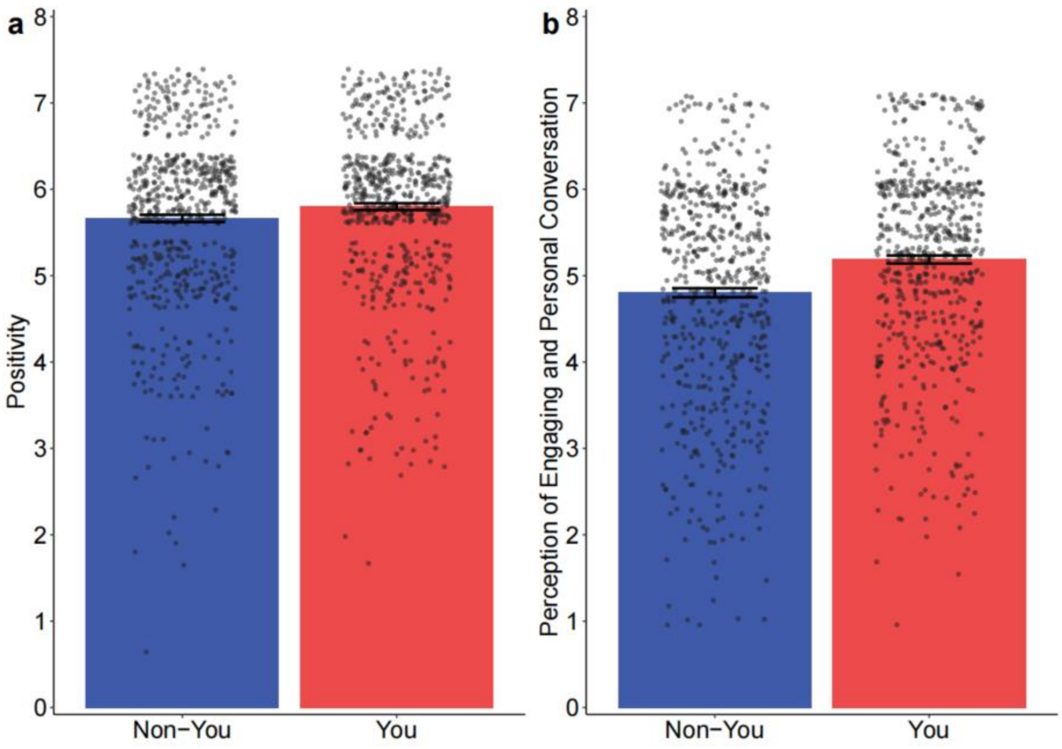

Supplementary Figure 10. Results of replication experiment. (**a**) Participants addressed with “you” (vs. non-“you”) rated the author’s response more positively. (**b**) Participants addressed with “you” (vs. non-“you”) found their conversation with the author more personal and engaging. Individual data points are shown using overlaid dot plots. Error bar shows ±1 SE.

Supplementary Tables

Supplementary Table 1. Total number of papers in each round of review

Review round	Total number of papers
1	13,359
2	12,320
3	3,320
4	102
5	43

Supplementary Table 2. Detailed description and summary statistics of four control variables

Variable Names	Measurement	Mean/Percentage	SD	Min	Max
Control Variable					
Last Initial of the First Author					
Start with A	Dummy variable takes value of 1 if the last initial of the first author of a paper is A.	0.035	0.184	0.000	1.000
Start with B	Dummy variable takes value of 1 if the last initial of the first author of a paper is B.	0.057	0.232	0.000	1.000
Start with C	Dummy variable takes value of 1 if the last initial of the first author of a paper is C.	0.064	0.244	0.000	1.000
Start with D	Dummy variable takes value of 1 if the last initial of the first author of a paper is D.	0.041	0.198	0.000	1.000
Start with E	Dummy variable takes value of 1 if the last initial of the first author of a paper is E.	0.011	0.105	0.000	1.000
Start with F	Dummy variable takes value of 1 if the last initial of the first author of a paper is F	0.029	0.169	0.000	1.000
Start with G	Dummy variable takes value of 1 if the last initial of the first author of a paper is G.	0.042	0.2	0.000	1.000
Start with H	Dummy variable takes value of 1 if the last initial of the first author of a paper is H.	0.054	0.226	0.000	1.000
Start with I	Dummy variable takes value of 1 if the last initial of the first author of a paper is I.	0.008	0.088	0.000	1.000
Start with J	Dummy variable takes value of 1 if the last initial of the first author of a paper is J.	0.023	0.151	0.000	1.000
Start with K	Dummy variable takes value of 1 if the last initial of the first author of a paper is K.	0.054	0.226	0.000	1.000
Start with L	Dummy variable takes value of 1 if the last initial of the first author of a paper is L	0.091	0.288	0.000	1.000
Start with M	Dummy variable takes value of 1 if the last initial of the first author of a paper is M.	0.069	0.253	0.000	1.000
Start with N	Dummy variable takes value of 1 if the last initial of the first author of a paper is N.	0.022	0.147	0.000	1.000
Start with O	Dummy variable takes value of 1 if the last initial of the first author of a paper is O.	0.014	0.116	0.000	1.000
Start with P	Dummy variable takes value of 1 if the last initial of the first author of a paper is P.	0.038	0.192	0.000	1.000
Start with Q	Dummy variable takes value of 1 if the last initial of the first author of a paper is Q.	0.006	0.075	0.000	1.000
Start with R	Dummy variable takes value of 1 if the last initial of the first author of a paper is R.	0.033	0.179	0.000	1.000
Start with S	Dummy variable takes value of 1 if the last initial of the first author of a paper is S.	0.093	0.29	0.000	1.000
Start with T	Dummy variable takes value of 1 if the last initial of the first author of a paper is T.	0.035	0.183	0.000	1.000
Start with U	Dummy variable takes value of 1 if the last initial of the first author of a paper is U.	0.003	0.056	0.000	1.000
Start with V	Dummy variable takes value of 1 if the last initial of the first author of a paper is V.	0.019	0.135	0.000	1.000
Start with W	Dummy variable takes value of 1 if the last initial of the first author of a paper is W.	0.059	0.236	0.000	1.000
Start with X	Dummy variable takes value of 1 if the last initial of the first author of a paper is X.	0.014	0.119	0.000	1.000
Start with Y	Dummy variable takes value of 1 if the last initial of the first author of a paper is Y.	0.033	0.18	0.000	1.000
Start with Z	Dummy variable takes value of 1 if the last initial of the first author of a paper is Z.	0.052	0.223	0.000	1.000
Month of Publication					
January	Dummy variable takes value of 1 if the publication month of a paper is January.	0.092	0.289	0.000	1.000

Variable Names	Measurement	Mean/Percentage	SD	Min	Max
February	Dummy variable takes value of 1 if the publication month of a paper is February.	0.08	0.272	0.000	1.000
March	Dummy variable takes value of 1 if the publication month of a paper is March.	0.075	0.264	0.000	1.000
April	Dummy variable takes value of 1 if the publication month of a paper is April.	0.076	0.265	0.000	1.000
May	Dummy variable takes value of 1 if the publication month of a paper is May.	0.08	0.272	0.000	1.000
June	Dummy variable takes value of 1 if the publication month of a paper is June.	0.082	0.274	0.000	1.000
July	Dummy variable takes value of 1 if the publication month of a paper is July.	0.081	0.272	0.000	1.000
August	Dummy variable takes value of 1 if the publication month of a paper is August.	0.08	0.272	0.000	1.000
September	Dummy variable takes value of 1 if the publication month of a paper is September.	0.087	0.282	0.000	1.000
October	Dummy variable takes value of 1 if the publication month of a paper is October.	0.099	0.298	0.000	1.000
November	Dummy variable takes value of 1 if the publication month of a paper is November.	0.098	0.297	0.000	1.000
December	Dummy variable takes value of 1 if the publication month of a paper is December.	0.07	0.255	0.000	1.000
Publication Year					
2016	Dummy variable takes value of 1 if the publication year of a paper is 2016.	0.05	0.219	0.000	1.000
2017	Dummy variable takes value of 1 if the publication year of a paper is 2017.	0.197	0.398	0.000	1.000
2018	Dummy variable takes value of 1 if the publication year of a paper is 2018.	0.242	0.428	0.000	1.000
2019	Dummy variable takes value of 1 if the publication year of a paper is 2019.	0.263	0.44	0.000	1.000
2020	Dummy variable takes value of 1 if the publication year of a paper is 2020.	0.233	0.423	0.000	1.000
2021	Dummy variable takes value of 1 if the publication year of a paper is 2021.	0.014	0.115	0.000	1.000
Paper Discipline*					
Biological Sciences	Dummy variable takes value of 1 if the discipline of a paper is biological sciences.	0.405	0.491	0.000	1.000
Physical Sciences	Dummy variable takes value of 1 if the discipline of a paper is physical sciences.	0.294	0.456	0.000	1.000
Health Sciences	Dummy variable takes value of 1 if the discipline of a paper is health sciences.	0.187	0.39	0.000	1.000
Earth and Environmental Sciences	Dummy variable takes value of 1 if the discipline of a paper is earth and environmental sciences.	0.099	0.298	0.000	1.000
Scientific Community and Society	Dummy variable takes value of 1 if the discipline of a paper is scientific community and society.	0.016	0.124	0.000	1.000

* The five official *Nature Communications* categories.

Supplementary Table 3. DID estimates with continuous usage of "you"

	(1) Number of Questions		(2) Number of Words		(3) Positivity (Python)		(4) Positivity (R)		(5) Negativity (Python)		(6) Negativity (Hand Coded)	
	Coef.	*t*-value	Coef.	*t*-value	Coef.	*t*-value	Coef.	*t*-value	Coef.	*t*-value	Coef.	*t*-value
Response with "You" [1, 2]	-2.5045***	-5.69	-97.3666***	-5.56	0.0057	1.88	0.0167***	3.40	-0.0009	-1.11	-0.0035***	-3.95
× After Response	(0.4399)		(17.5068)		(0.0030)		(0.0049)		(0.0008)		(0.0009)	
Response with "You" [3, 5]	-2.6939***	-3.14	-115.3332***	-3.39	0.0053	0.92	0.0133	1.52	-0.0010	-0.71	-0.0061***	-3.68
× After Response	(0.8578)		(34.0645)		(0.0058)		(0.0088)		(0.0014)		(0.0017)	
Response with "You" [6, max]	-5.5735***	-8.33	-211.3986***	-7.77	0.0009	0.21	0.0217***	3.33	-0.0043***	-3.99	-0.0069***	-5.36
× After Response	(0.6688)		(27.2203)		(0.0040)		(0.0065)		(0.0011)		(0.0013)	
After Response	-21.5962***	-111.77	-1227.1963***	-148.06	0.0515***	36.07	0.0685***	29.77	-0.0265***	-62.64	-0.0319***	-80.67
	(0.1932)		(8.2887)		(0.0014)		(0.0023)		(0.0004)		(0.0004)	
Control Variables	Yes		Yes		Yes		Yes		Yes		Yes	
Paper Fixed Effects	Yes		Yes		Yes		Yes		Yes		Yes	
Observations	24,640		24,640		24,640		24,640		24,640		24,640	
R^2	0.524		0.662		0.128		0.110		0.288		0.411	

Notes: Each column in the table represents a DID regression with control variables and paper fixed effects. The coefficients of the variable Response with "You" [1, 2], Response with "You" [3, 5], and Response with "You" [6, max] are not included in this table, in that the pure effect of "you" usage is absorbed by paper fixed effects. Standard errors in parentheses are clustered at the paper level. * $p < 0.05$, ** $p < 0.01$, *** $p < 0.001$. Two-sided *t* tests with a 95% confidence interval are employed here and throughout the tables of Supplementary Information.

Supplementary Table 4. DID estimates with treatment group excluding courteous usage of "you"

	(1) Positivity (Python)		(2) Positivity (R)		(3) Negativity (Python)		(4) Negativity (Hand Coded)	
	Coef.	*t*-value	Coef.	*t*-value	Coef.	*t*-value	Coef.	*t*-value
Response with "You"	0.0067*	2.37	0.0190***	4.20	-0.0021**	-2.70	-0.0038***	-4.58
× After Response	(0.0028)		(0.0045)		(0.0008)		(0.0008)	
After Response	0.0510***	34.56	0.0679***	28.45	-0.0264***	-59.68	-0.0317***	-78.10
	(0.0015)		(0.0024)		(0.0004)		(0.0004)	
Control Variables	Yes		Yes		Yes		Yes	
Paper Fixed Effects	Yes		Yes		Yes		Yes	
Observations	21,168		21,168		21,168		21,168	
R^2	0.127		0.108		0.282		0.406	

Notes: Each column in the table represents a DID regression with control variables and paper fixed effects. The coefficients of the variable Response with "You" are not included in this table, in that the pure effect of "you" usage is absorbed by paper fixed effects. Standard errors in parentheses are clustered at the paper level. * $p < 0.05$, ** $p < 0.01$, *** $p < 0.001$.

Supplementary Table 5. DID estimates with the usage of "the reviewer" as control group

	(1) Positivity (Python)		(2) Positivity (R)		(3) Negativity (Python)		(4) Negativity (Hand Coded)	
	Coef.	*t*-value	Coef.	*t*-value	Coef.	*t*-value	Coef.	*t*-value
Response with "You"	0.0050*	2.00	0.0135***	3.35	-0.0014*	-2.04	-0.0039***	-5.27
× After Response	(0.0025)		(0.0040)		(0.0007)		(0.0007)	
After Response	0.0511***	31.69	0.0716***	27.18	-0.0269***	-55.42	-0.0327***	-73.39
	(0.0016)		(0.0026)		(0.0005)		(0.0004)	
Control Variables	Yes		Yes		Yes		Yes	
Paper Fixed Effects	Yes		Yes		Yes		Yes	
Observations	21,956		21,956		21,956		21,956	
R^2	0.129		0.113		0.294		0.420	

Notes: Each column in the table represents a DID regression with control variables and paper fixed effects. The coefficients of the variable Response with "You" are not included in this table, in that the pure effect of "you" usage is absorbed by paper fixed effects. Standard errors in parentheses are clustered at the paper level. * $p < 0.05$, ** $p < 0.01$, *** $p < 0.001$.

Supplementary Table 6. Mean differences before and after matching

	Unmatched (U) Matched (M)	Mean Treated	Mean Control	%bias	*t*-value	p-value
Number of Pages	U	11.349	11.240	3.5	2.66	0.008
	M	11.348	11.460	-3.6	-2.50	0.012
Number of References	U	57.705	56.084	10.1	7.76	0.000
	M	57.704	57.216	3.0	2.08	0.038
Title Length	U	11.477	11.397	2.9	2.21	0.027
	M	11.478	11.499	-0.7	-0.52	0.606
Number of Authors	U	10.488	10.589	-0.8	-0.58	0.560
	M	10.489	10.346	1.1	0.78	0.436
H-index of the First Author	U	15.135	14.485	5.3	4.03	0.000
	M	15.136	15.039	0.8	0.52	0.604
Gender of the First Author	U	0.305	0.307	-0.4	-0.32	0.751
	M	0.305	0.308	-0.6	-0.41	0.683
Last Initial of the First Author						
Start with A	U	0.036	0.035	0.8	0.63	0.530
	M	0.036	0.039	-1.6	-1.07	0.286
Start with B	U	0.053	0.060	-3.4	-2.54	0.011
	M	0.053	0.053	-0.1	-0.06	0.948
Start with C	U	0.065	0.063	0.7	0.56	0.574
	M	0.065	0.063	0.8	0.53	0.595
Start with D	U	0.036	0.044	-4.0	-3.01	0.003
	M	0.036	0.041	-2.2	-1.58	0.114
Start with E	U	0.010	0.012	-1.9	-1.41	0.158
	M	0.010	0.010	-0.6	-0.43	0.665
Start with F	U	0.028	0.030	-1.4	-1.04	0.298
	M	0.028	0.029	-0.7	-0.52	0.602
Start with G	U	0.039	0.044	-2.4	-1.85	0.064
	M	0.039	0.038	0.6	0.45	0.652
Start with H	U	0.060	0.050	4.1	3.15	0.002
	M	0.060	0.056	1.7	1.18	0.238
Start with I	U	0.009	0.007	2.0	1.57	0.118
	M	0.009	0.009	0.0	0.00	1.000
Start with J	U	0.022	0.024	-1.2	-0.92	0.357
	M	0.022	0.021	0.4	0.30	0.766
Start with K	U	0.056	0.052	1.6	1.21	0.228
	M	0.056	0.060	-1.9	-1.24	0.215
Start with L	U	0.088	0.093	-1.6	-1.24	0.216
	M	0.088	0.086	0.7	0.46	0.644
Start with M	U	0.071	0.067	1.3	1.03	0.302
	M	0.071	0.070	0.2	0.17	0.865
Start with N	U	0.023	0.022	0.9	0.66	0.506
	M	0.023	0.022	0.6	0.39	0.696
Start with O	U	0.013	0.014	-0.6	-0.46	0.649
	M	0.013	0.013	0.5	0.39	0.700
Start with P	U	0.035	0.040	-2.9	-2.17	0.030
	M	0.035	0.035	0.1	0.08	0.937
Start with Q	U	0.005	0.006	-0.1	-0.11	0.911

	Unmatched (U) Matched (M)	Mean Treated	Mean Control	%bias	*t*-value	p-value
	M	0.005	0.005	0.6	0.40	0.688
Start with R	U	0.026	0.038	-6.9	-5.16	0.000
	M	0.026	0.026	-0.1	-0.09	0.927
Start with S	U	0.096	0.091	2.0	1.50	0.133
	M	0.096	0.099	-1.0	-0.68	0.495
Start with T	U	0.035	0.034	0.4	0.31	0.760
	M	0.035	0.035	0.1	0.08	0.937
Start with U	U	0.003	0.003	-1.1	-0.81	0.421
	M	0.003	0.003	0.0	0.00	1.000
Start with V	U	0.016	0.020	-3.4	-2.57	0.010
	M	0.016	0.015	0.5	0.35	0.724
Start with W	U	0.060	0.058	0.8	0.58	0.560
	M	0.060	0.059	0.4	0.24	0.807
Start with X	U	0.017	0.013	3.6	2.78	0.005
	M	0.017	0.017	-0.2	-0.11	0.912
Start with Y	U	0.040	0.029	6.2	4.86	0.000
	M	0.040	0.039	0.7	0.45	0.655
Start with Z	U	0.058	0.050	3.5	2.70	0.007
	M	0.058	0.056	0.7	0.50	0.617
Positivity of Authors (1st Round)	U	0.088	0.086	6.8	5.04	0.000
	M	0.088	0.089	-1.0	-0.76	0.450
Friendly of Authors (1st Round)	U	0.098	0.099	-2.1	-1.60	0.110
	M	0.098	0.098	0.7	0.52	0.602
Positivity of Reviewers (1st Round)	U	0.322	0.263	27.3	21.34	0.000
	M	0.320	0.302	8.5	5.72	0.000
Month of Publication						
January	U	0.094	0.092	0.6	0.45	0.650
	M	0.094	0.096	-0.9	-0.64	0.521
February	U	0.077	0.082	-1.9	-1.43	0.152
	M	0.077	0.077	0.1	0.05	0.957
March	U	0.078	0.074	1.4	1.08	0.280
	M	0.078	0.077	0.3	0.22	0.829
April	U	0.076	0.075	0.3	0.25	0.803
	M	0.076	0.078	-0.5	-0.33	0.744
May	U	0.077	0.082	-2.0	-1.49	0.136
	M	0.077	0.076	0.1	0.05	0.957
June	U	0.078	0.084	-2.4	-1.80	0.072
	M	0.078	0.079	-0.3	-0.22	0.829
July	U	0.084	0.078	2.1	1.64	0.101
	M	0.084	0.084	0.1	0.05	0.958
August	U	0.079	0.080	-0.3	-0.26	0.792
	M	0.079	0.079	-0.1	-0.05	0.957
September	U	0.086	0.088	-0.6	-0.49	0.625
	M	0.086	0.087	-0.3	-0.21	0.837
October	U	0.099	0.098	0.2	0.19	0.849
	M	0.099	0.100	-0.4	-0.24	0.809
November	U	0.102	0.096	2.2	1.70	0.089

	Unmatched (U) Matched (M)	Mean Treated	Mean Control	%bias	*t*-value	p-value
	M	0.102	0.097	1.9	1.31	0.191
December	U	0.070	0.070	0.0	0.02	0.986
	M	0.070	0.070	-0.1	-0.06	0.955
Publication Year						
2016	U	0.038	0.053	-7.6	-5.71	0.000
	M	0.038	0.035	1.3	1.01	0.313
2017	U	0.165	0.216	-13.1	-9.87	0.000
	M	0.165	0.170	-1.3	-0.93	0.352
2018	U	0.235	0.249	-3.4	-2.59	0.010
	M	0.235	0.229	1.4	0.96	0.336
2019	U	0.278	0.259	4.4	3.41	0.001
	M	0.278	0.282	-0.9	-0.61	0.540
2020	U	0.267	0.211	13.1	10.11	0.000
	M	0.267	0.267	-0.1	-0.07	0.948
2021	U	0.017	0.011	5.1	3.96	0.000
	M	0.017	0.017	0.7	0.45	0.654
Paper Discipline						
Biological Sciences	U	0.374	0.421	-9.5	-7.25	0.000
	M	0.375	0.405	-6.1	-4.25	0.000
Physical Sciences	U	0.249	0.320	-15.7	-11.89	0.000
	M	0.249	0.258	-1.9	-1.37	0.172
Health Sciences	U	0.202	0.180	5.7	4.36	0.000
	M	0.202	0.213	-2.8	-1.89	0.058
Earth and Environmental Sciences	U	0.150	0.068	26.3	20.91	0.000
	M	0.150	0.107	13.7	8.76	0.000
Scientific Community and Society	U	0.024	0.011	10.2	8.19	0.000
	M	0.024	0.017	5.6	3.58	0.000

Notes: The PSM matching results are based on the measurement of Positivity (Python). The 1:1 nearest neighbor matching algorithm is employed. After matching, both the treatment and control groups have a sample size of 9,532. The *p*-values are obtained from two-sided *t*-tests with a 95% confidence interval.

Supplementary Table 7. Estimation results with PSM-DID approach

	(1) Positivity (Python)		(2) Positivity (R)		(3) Negativity (Python)		(4) Negativity (Hand Coded)	
	Coef.	*t*-value	Coef.	*t*-value	Coef.	*t*-value	Coef.	*t*-value
Response with "You"	0.0036	1.37	0.0103*	2.38	-0.0014	-1.83	-0.0031***	-3.92
× After Response	(0.0026)		(0.0043)		(0.0008)		(0.0008)	
After Response	0.0525***	29.23	0.0749***	24.69	-0.0270***	-47.53	-0.0335***	-63.82
	(0.0018)		(0.0030)		(0.0006)		(0.0005)	
Control Variables	Yes		Yes		Yes		Yes	
Paper Fixed Effects	Yes		Yes		Yes		Yes	
Observations	19,064		19,064		19,064		19,064	
R^2	0.132		0.118		0.296		0.414	

Notes: Each column in the table represents a PSM-DID regression with control variables and paper fixed effects. The coefficients of the variable Response with "You" are not included in this table, in that the pure effect of "you" usage is absorbed by paper fixed effects. Standard errors are reported in parentheses and are clustered at the paper level. * $p < 0.05$, ** $p < 0.01$, *** $p < 0.001$.

Supplementary Table 8. Estimation results with Heckman model (first stage)

	Response with "You"	
	Coef.	*t*-value
Reviewers' usage of first-person pronoun (1st review round)	0.3756***	20.91
	(0.0180)	
Gender of the First Author	-0.0018	-0.10
	(0.0179)	
H-index of the First Author	0.0017***	2.60
	(0.0007)	
Control Variables	Yes	
Observations	25,679	

Notes: Standard errors in parentheses are clustered at the paper level. * $p < 0.05$, ** $p < 0.01$, *** $p < 0.001$.

Supplementary Table 9. Estimation results with Heckman model (second stage)

	(1)		(2)		(3)		(4)	
	Positivity (Python)		Positivity (R)		Negativity (Python)		Negativity (Hand Coded)	
	Coef.	*t*-value	Coef.	*t*-value	Coef.	*t*-value	Coef.	*t*-value
Response with "You"	0.0051*	2.14	0.0187***	4.76	-0.0023***	-3.32	-0.0054***	-7.31
× After Response	(0.0024)		(0.0039)		(0.0007)		(0.0007)	
Response with "You"	0.0006	1.65	-0.0035***	-2.97	0.0006	1.62	0.0048***	7.49
	(0.0004)		(0.0012)		(0.0004)		(0.0006)	
After Response	0.0510***	34.61	0.0651***	26.95	-0.0257***	-55.44	-0.0305***	-73.13
	(0.0015)		(0.0024)		(0.0005)		(0.0004)	
IMR	-0.0061	-1.25	-0.0323***	-4.01	-0.0033*	-2.19	-0.0214***	-13.28
	(0.0049)		(0.0081)		(0.0015)		(0.0016)	
Control Variables	Yes		Yes		Yes		Yes	
Paper Fixed Effects	No		No		No		No	
Observations	25,679		25,679		25,679		25,679	
R^2	0.153		0.084		0.203		0.319	

Notes: Each column in the table represents a Heckman regression with control variables and paper fixed effects. The results still exist with paper fixed effects included. Standard errors in parentheses are clustered at the paper level. * $p < 0.05$, ** $p < 0.01$, *** $p < 0.001$.

Supplementary Table 10. Qualitative evidence of some examples with more and less subjective sense

Subjectivity scores	Paper Title	DOI	Examples
0.75	Low excitatory innervation balances high intrinsic excitability of immature dentate neurons	10.1038/ncomms11313	The revised version of the manuscript has been substantially improved and it seems now suitable for publication.
0.53	Rapid and high-resolution patterning of microstructure and composition in organic semiconductors using ‘molecular gates’	10.1038/s41467-020-17361-8	The paper is novel, and broadly interesting, and I would likely recommend for publication after a few clarifications.
0.6	Mummified precocial bird wings in mid-Cretaceous Burmese amber	10.1038/ncomms12089	This interesting manuscript describes two partial wings with plumage, exquisitely preserved in mid-Cretaceous amber from Myanmar.
0.2	Killer-like receptors and GPR56 progressive expression defines cytokine production of human CD4+ memory T cells	10.1038/s41467-019-10018-1	In this manuscript Truong et. al. put forth an alternative classification scheme for CD4+ memory T cells based on surface markers related to cytokine production instead of the classical TCM, TEM, TEMRA divisions.
0.1	Schistosoma mansoni treatment reduces HIV entry into cervical CD4+ T cells and induces IFN-I pathways	10.1038/s41467-019-09900-9	The investigators report that, following treatment of S. mansoni infection, women had induction of genital and systemic circulation IFN-I pathways, suggesting an increase in anti-viral immunity.

Supplementary Table 11. Qualitative evidence of some examples with complex and simple words

Complex Word scores	Paper Title	DOI	Examples
2.48	Legionella effector MavC targets the Ube2N~Ub conjugate for noncanonical ubiquitination	10.1038/s41467-020-16211-x	The manuscript is an insightful contribution to our understanding of how bacterial effectors target Ub signaling pathways and elucidates how MavC favors Ub transglutamination over deamidation activity.
2.35	The UbiX flavin prenyltransferase reaction mechanism resembles class I terpene cyclase chemistry	10.1038/s41467-019-10220-1	The chemistry is interesting and the experiments employ a large number of complementary techniques; activity, site-directed mutagenesis, UV-vis and EPR spectroscopy, and crystallography.
2.26	Probing a battery electrolyte drop with ambient pressure photoelectron spectroscopy	10.1038/s41467-019-10803-y	This is an interesting battery related surface science work with unique procedure of using PC solvent vapor pressure instead of traditional Ar partial pressure for AP-XPS measurements
1.75	10.1038/s41467-021-21773-5	10.1038/s41467-021-21773-5	I enjoyed reading this manuscript and think that it is a good piece of work that is going in the right direction to better understand SH climate variability.
1.62	An integrated genomic regulatory network of virulence-related transcriptional factors in Pseudomonas aeruginosa	10.1038/s41467-019-10778-w	I think that this data will be useful to the field and this manuscript brings together and extends much useful data on virulence in Pa.
1.61	Transplantation of discarded livers following viability testing with normothermic machine perfusion	10.1038/s41467-020-16251-3	I believe this to be a critical point for the authors to include in the manuscript proper and am certain it will be of interest to those who read this work.

Supplementary Table 12. Different use of singular and plural first-person pronouns by reviewers

	(1) Reviewers' Plural First-person Pronouns		(2) Reviewers' Singular First-person Pronouns	
	Coef.	*t*-value	Coef.	*t*-value
Response with "You"	-0.2424	-1.57	-1.0918***	-6.84
× After Response	(0.1544)		(0.1596)	
After Response	-0.0312	-0.39	-2.9931***	-35.56
	(0.0793)		(0.0842)	
Control Variables	Yes		Yes	
Paper Fixed Effects	Yes		Yes	
Observations	24,640		24,640	
R^2	0.049		0.522	

Notes: Each column in the table represents a DID regression with control variables and paper fixed effects. The coefficients of the variable Response with "You" are not included in this table, in that the pure effect of "you" usage is absorbed by paper fixed effects. Standard errors are reported in parentheses and are clustered at the paper level. * $p < 0.05$, ** $p < 0.01$, *** $p < 0.001$.

Supplementary Table 13. Estimation results with alternative number of topics

	(1)		(2)		(3)		(4)	
	Topics = 35		Topics = 45		"High-engagement" Words		STM Model	
	Coef.	*t*-value	Coef.	*t*-value	Coef.	*t*-value	Coef.	*t*-value
Response with "You"	0.0991***	4.33	0.1092***	4.29	0.2675	1.14	0.0210	1.38
× After Response	(0.0229)		(0.0255)		(0.2337)		(0.0152)	
After Response	0.5859***	41.14	0.7673***	48.42	-5.1249***	-37.13	0.1623***	16.73
	(0.0142)		(0.0158)		(0.1380)		(0.0097)	
Control Variables	Yes		Yes		Yes		Yes	
Paper Fixed Effects	Yes		Yes		Yes		Yes	
Observations	24,640		24,640		24,640		24,640	
R^2	0.203		0.193		0.358		0.301	

Notes: Each column in the table represents a DID regression with control variables and paper fixed effects. The coefficients of the variable Response with "You" are not included in this table, in that the pure effect of "you" usage is absorbed by paper fixed effects. Standard errors are reported in parentheses and are clustered at the paper level. * $p < 0.05$, ** $p < 0.01$, *** $p < 0.001$.

Supplementary Table 14. Estimation results with alternative topics in the latent 40 topics

	(1) Topic18: Exposition		(2) Topic36: Methodology		(3) Topic13: Electromagnetism		(4) Topic26: Ecology	
	Coef.	*t*-value	Coef.	*t*-value	Coef.	*t*-value	Coef.	*t*-value
Response with "You"	0.0009	0.03	0.0256	1.13	0.0127	0.76	-0.0021	-0.09
× After Response	(0.0283)		(0.0227)		(0.0168)		(0.0249)	
After Response	-1.4491***	-83.58	-0.9158***	-66.95	-0.5999***	-58.35	-0.9930***	-66.88
	(0.0173)		(0.0137)		(0.0103)		(0.0148)	
Control Variables	Yes		Yes		Yes		Yes	
Paper Fixed Effects	Yes		Yes		Yes		Yes	
Observations	24,640		24,640		24640		24640	
R^2	0.518		0.511		0.506		0.479	

Notes: Each column in the table represents a DID regression with control variables and paper fixed effects. The coefficients of the variable Response with "You" are not included in this table, in that the pure effect of "you" usage is absorbed by paper fixed effects. Standard errors are reported in parentheses and are clustered at the paper level. * $p < 0.05$, ** $p < 0.01$, *** $p < 0.001$.

Supplementary Table 15. Vocabulary for calculating the frequency of negative words, "high-engagement" words, and friendly words used by authors in 1st review round

Variables	Words of Dictionaries for calculating the variables	Number of Words
Negativity (Hand Coded)	abnormal, absurd, adverse, ambivalent, arcane, baffling, bias, biased, complex, concerned, contradictory, deficient, difficult, disturbing, erroneous, faulty, flawed, foolish, fraught, fulsome, fuzzy, groundless, hard, illogical, impenetrable, implausible, impossible, inaccurate, inadequate, incoherent, incomplete, inconclusive, inconsiderable, inconsistent, inconvincible, indefensible, ineffective, inexpert, infeasible, invalid, irrelevant, irresponsible, lacking, limited, limiting, misguided, missing, mistaken, needless, negative, objectionable, poor, poorly, questionable, repellent, repulsive, risky, serious, severe, shocking, stupid, terrible, troublesome, unable, unacceptable, unattractive, unbelievable, uncertain, uncharacteristic, unclear, unconvinced, unconvincing, unethical, unfavorable, uninterpretable, unknown, unmoral, unpractical, unpredictable, unprofessional, unreadable, unreal, unrealistic, unreliable, unrepresentative, unsatisfactory, unsuitable, unsupported, untraceable, unwelcome, worrisome, worst	92
"High-engagement" Words	absorbing, address, agreeable, allure, alluring, appealing, appreciate, arresting, attach, attract, attracting, attraction, attractive, be a party to, be absorbed in, be associated with, become involved in, captivate, captivating, captivation, charismatic, charm, charming, collaborate, concentrated upon, concern, concerned with, congenial, cooperate, cute, delightful, embark on, enamour, enchant, enchanting, enchantment, endearing, engage, engaged, engaging, engross, engrossed, engrossing, engulf, enjoy, enjoyable, entertaining , enticing, entrancing, enwrap, espousal, exciting, fascinate, fascinating, fill up, get involved in, glamorous, go in for, gorgeous, gratifying, heavenly, immerse, improve, interest, interesting, intrigue, intriguing, inviting, involve, involvement, likable, likeable, lovable, lovely, magnetic, mesmeric, mesmerizing, now, obsess, obsessed, obsession, occupying, partake, partake in, partake of, participate in, participation, play a part in, play a role in, pleasant, pleasing, pleasurable, point, preoccupy, prepossessing, pretty, previous, provocative, raise, recommend, response, revise, satisfying, seductive, set about, share, share in, siren, smashing, still, sweet, take on, take part in, take upon oneself, tempting, version	116
Friendliness of Authors (1st Round)	acceptable, accommodating, accommodative, accurate, actionable, adequate, advanced, advantageous, affable, affectionate, agreeable, amenable, amiable, amicable, apparent, appreciative, attractive, beneficial, benevolent, benign, better, bright, broad, broad-minded, capable, chirpy, chummy, clear, clubbable, clubby, communicative, companionable, companionate, complaisant, complete, compliant, comprehensive, conciliatory, conclusive, confident, confiding, congenial, constructive, conversable, convincing, convivial, cordial, correct, courteous, critical, crucial, dear, decisive, deep, deliberate, desired, devoted,	178

Variables	Words of Dictionaries for calculating the variables	Number of Words
	distinct, easy-going, effective, efficient, energetic, essential, evident, excellent, exciting, expecting, famous, fantastic, favorable, favored, flexible, forgiving, forthcoming, fraternal, fresh, friendliness, friendly, fruitful, fundamental, genial, gentle, glad, good, gracious, grateful, great, greater, gregarious, harmonious, healthy, hearty, helpful, hospitable, important, impressive, indispensable, influential, informative, insightful, interested, interesting, jolly, jovial, kind, kind-hearted, kindly, lovable, lovesome, loving, massive, meaningful, natural, neighborly, nice, non-hostile, notable, noteworthy, optimal, peaceable, perfect, persistent, plausible, popular, positive, powerful, precious, precise, productive, profound, prominent, pronounced, proper, reasonable, reproducible, right, rigorous, robust, satisfactory, seminal, sensible, significant, sincere, sizable, smooth, solid, special, steady, straightforward, striking, strong, successful, sufficient, suitable, supporting, sustainable, tender-hearted, thoughtful, trustworthy, unceremonious, understandable, unique, unprecedented, unreserved, useful, valuable, valued, versatile, vigorous, vital, warm-hearted, welcoming, well-disposed, wise, wonderful, worth, worthwhile, worthy	

Supplementary Table 16. Estimates for a "you" conversation initiated by reviewers

	(1) Subjectivity		(2) First-person Singular Pronouns Usage		(3) Word Complexity		(4) Reviewer Engagement	
	Coef.	*t*-value	Coef.	*t*-value	Coef.	*t*-value	Coef.	*t*-value
Response with "You"	0.0045	1.17	-1.1686***	-3.65	-0.0193***	-4.01	0.1417***	4.65
× After Response	(0.0038)		(0.3204)		(0.0048)		(0.0305)	
After Response	0.0243***	8.96	-5.4837***	-27.20	0.0335***	11.11	-0.7065***	-31.07
	(0.0027)		(0.2016)		(0.0030)		(0.0227)	
Control Variables	YES		YES		YES		YES	
Paper Fixed Effects	YES		YES		YES		YES	
Observations	8,278		8,278		8,278		8,278	
R^2	0.067		0.528		0.205		0.441	

Notes: Each column in the table represents a DID regression with control variables and paper fixed effects. The coefficients of the variable Response with "You" are not included in this table, in that the pure effect of "you" usage is absorbed by paper fixed effects. Standard errors are reported in parentheses and are clustered at the paper level. * $p < 0.05$, ** $p < 0.01$, *** $p < 0.001$.

Supplementary Table 17. Estimates for a "you" conversation not initiated by reviewers

	(1) Subjectivity		(2) First-person Singular Pronouns Usage		(3) Word Complexity		(4) Reviewer Engagement	
	Coef.	*t*-value	Coef.	*t*-value	Coef.	*t*-value	Coef.	*t*-value
Response with "You" × After Response	0.0019	0.61	0.0336	0.22	-0.0049	-1.25	0.0613*	2.56
	(0.0032)		(0.1541)		(0.0039)		(0.0239)	
After Response	0.0176***	9.51	-2.0787***	-24.50	0.0486***	19.84	-0.7693***	-55.66
	(0.0018)		(0.0849)		(0.0024)		(0.0138)	
Control Variables	YES		YES		YES		YES	
Paper Fixed Effects	YES		YES		YES		YES	
Observations	16,362		16,362		16,362		16,362	
R^2	0.045		0.478		0.207		0.506	

Notes: Each column in the table represents a DID regression with control variables and paper fixed effects. The coefficients of the variable Response with "You" are not included in this table, in that the pure effect of "you" usage is absorbed by paper fixed effects. Standard errors are reported in parentheses and are clustered at the paper level. * $p < 0.05$, ** $p < 0.01$, *** $p < 0.001$.

Supplementary Table 18. Estimates for a "you" conversation initiated by reviewers on six behavioral outcomes

	(1) Number of Questions		(2) Number of Words		(3) Positivity (Python)		(4) Positivity (R)		(5) Negativity (Python)		(6) Negativity (Hand Coded)	
	Coef.	*t*-value	Coef.	*t*-value	Coef.	*t*-value	Coef.	*t*-value	Coef.	*t*-value	Coef.	*t*-value
Response with "You"	-2.8739***	-4.19	-105.1980***	-4.01	0.0135***	3.39	0.0178**	2.71	-0.0028*	-2.54	-0.0051***	-3.95
× After Response	(0.6853)		(26.2322)		(0.0040)		(0.0066)		(0.0011)		(0.0013)	
After Response	-27.5335***	-61.47	-1445.4813***	-79.99	0.0478***	18.04	0.0794***	17.28	-0.0254***	-30.26	-0.0370***	-43.96
	(0.4480)		(18.0717)		(0.0026)		(0.0046)		(0.0008)		(0.0008)	
Control Variables	YES		YES		YES		YES		YES		YES	
Paper Fixed Effects	YES		YES		YES		YES		YES		YES	
Observations	8,278		8,278		8,278		8,278		8,278		8,278	
R^2	0.546		0.688		0.132		0.131		0.306		0.426	

Notes: Each column in the table represents a DID regression with control variables and paper fixed effects. The coefficients of the variable Response with "You" are not included in this table, in that the pure effect of "you" usage is absorbed by paper fixed effects. Standard errors are reported in parentheses and are clustered at the paper level. * $p < 0.05$, ** $p < 0.01$, *** $p < 0.001$.

Supplementary Table 19. Estimates for a "you" conversation not initiated by reviewers on six behavioral outcomes

	(1) Number of Questions		(2) Number of Words		(3) Positivity (Python)		(4) Positivity (R)		(5) Negativity (Python)		(6) Negativity (Hand Coded)	
	Coef.	*t*-value	Coef.	*t*-value	Coef.	*t*-value	Coef.	*t*-value	Coef.	*t*-value	Coef.	*t*-value
Response with "You"	-1.1120**	-3.02	-63.3841***	-3.79	-0.0002	-0.07	0.0119*	2.44	-0.0016	-1.90	-0.0024**	-2.89
× After Response	(0.3685)		(16.7345)		(0.0030)		(0.0049)		(0.0009)		(0.0008)	
After Response	-19.2448***	-92.68	-1137.2761***	-121.54	0.0522***	29.52	0.0636***	22.82	-0.0268***	-51.45	-0.0298***	-64.94
	(0.2076)		(9.3569)		(0.0018)		(0.0028)		(0.0005)		(0.0005)	
Control Variables	YES		YES		YES		YES		YES		YES	
Paper Fixed Effects	YES		YES		YES		YES		YES		YES	
Observations	16,362		16,362		16,362		16,362		16,362		16,362	
R^2	0.518		0.649		0.127		0.0970		0.279		0.398	

Notes: Each column in the table represents a DID regression with control variables and paper fixed effects. The coefficients of the variable Response with "You" are not included in this table, in that the pure effect of "you" usage is absorbed by paper fixed effects. Standard errors are reported in parentheses and are clustered at the paper level. * $p < 0.05$, ** $p < 0.01$, *** $p < 0.001$.

Supplementary References

1. Heckman, J. J. Sample selection bias as a specification error. *Econom. J. Econom. Soc.* 153–161 (1979).
2. Leung, F. F., Gu, F. F., Li, Y., Zhang, J. Z. & Palmatier, R. W. Influencer marketing effectiveness. *J. Mark.* **86**, 93–115 (2022).
3. Su, L., Sengupta, J., Li, Y. & Chen, F. “Want” versus “Need”: How linguistic framing influences responses to crowdfunding appeals. *J. Consum. Res.* ucad033 (2023).
4. Si, K., Li, Y., Ma, C. & Guo, F. Affiliation bias in peer review and the gender gap. *Res. Policy* **52**, 104797 (2023).
5. Sun, Z., Liu, S., Li, Y. & Ma, C. Expedited editorial decision in COVID-19 pandemic. *J. Informetr.* **17**, 101382 (2023).
6. Bravo, G., Grimaldo, F., López-Iñesta, E., Mehmani, B. & Squazzoni, F. The effect of publishing peer review reports on referee behavior in five scholarly journals. *Nat. Commun.* **10**, 1–8 (2019).
