# Supplementary material for: Behavioral consequences of second-person pronouns in written communications between authors and reviewers of scientific papers
Source: Nat Commun. 2024 Jan 2;15:152. doi: 10.1038/s41467-023-44515-1 (PMC10762116; doi:10.1038/s41467-023-44515-1)
Supplement: Supplementary file 3 — Reporting Summary [file 41467_2023_44515_MOESM3_ESM.pdf]

nature portfolio

Corresponding author(s): NCOMMS-22-39574-T

Last updated by author(s): Dec 2, 2023

Reporting Summary

Nature Portfolio wishes to improve the reproducibility of the work that we publish. This form provides structure for consistency and transparency in reporting. For further information on Nature Portfolio policies, see our Editorial Policies and the Editorial Policy Checklist.

Statistics

For all statistical analyses, confirm that the following items are present in the figure legend, table legend, main text, or Methods section.

n/a	Confirmed	
☐	☒	The exact sample size (n) for each experimental group/condition, given as a discrete number and unit of measurement
☐	☒	A statement on whether measurements were taken from distinct samples or whether the same sample was measured repeatedly
☐	☒	The statistical test(s) used AND whether they are one- or two-sided<br>*Only common tests should be described solely by name; describe more complex techniques in the Methods section.*
☐	☒	A description of all covariates tested
☐	☒	A description of any assumptions or corrections, such as tests of normality and adjustment for multiple comparisons
☐	☒	A full description of the statistical parameters including central tendency (e.g. means) or other basic estimates (e.g. regression coefficient) AND variation (e.g. standard deviation) or associated estimates of uncertainty (e.g. confidence intervals)
☐	☒	For null hypothesis testing, the test statistic (e.g. F, t, r) with confidence intervals, effect sizes, degrees of freedom and P value noted<br>*Give P values as exact values whenever suitable.*
☒	☐	For Bayesian analysis, information on the choice of priors and Markov chain Monte Carlo settings
☒	☐	For hierarchical and complex designs, identification of the appropriate level for tests and full reporting of outcomes
☐	☒	Estimates of effect sizes (e.g. Cohen's d, Pearson's r), indicating how they were calculated

Our web collection on statistics for biologists contains articles on many of the points above.

Software and code

Policy information about availability of computer code

Data collection	We collected revision correspondence of 13,359 papers published in Nature Communications between April 2016 (when the journal first began publishing reviewer reports) and April 2021 using Python (version 3.7).<br><br>Behavioral experiments were designed employing online survey maker survey Qualtrics. The survey was subsequently distributed to Amazon Mechanical Turk (MTurk) panelists via the CloudResearch platform (formerly unknown as TurkPrime). The data are hosted on and retrieved from Qualtrics.
Data analysis	Python (version 3.7) was used for the following tasks:<br>1. Scrawling peer review data;<br>2. Calculating the following variables: “You” Usage, Number of Questions, Number of Words, Positivity (Python), Negativity (Python), Negativity (Hand Coded), Subjectivity, First-person Singular Pronoun Usage, Word Complexity;<br>3. Calculating the following control variables: Positivity of Authors (1st Round), Friendliness of Authors (1st Round), Positivity of Reviewers (1st Round).<br><br>R Studio (version 4.1.2) was used for the following tasks:<br>1. Calculating the following variable: Positivity (R);<br>2. Analyzing data from behavioral experiments in the main text and in the SI;<br>3. Producing graphs for behavioral experiments in the main text and in the SI.<br><br>Stata (version 14.1) was used for the following tasks:

1. All remaining data analyses;
2. All remaining graph production.

For manuscripts utilizing custom algorithms or software that are central to the research but not yet described in published literature, software must be made available to editors and reviewers. We strongly encourage code deposition in a community repository (e.g. GitHub). See the Nature Portfolio guidelines for submitting code & software for further information.

Data

Policy information about availability of data

All manuscripts must include a data availability statement. This statement should provide the following information, where applicable:

- Accession codes, unique identifiers, or web links for publicly available datasets
- A description of any restrictions on data availability
- For clinical datasets or third party data, please ensure that the statement adheres to our policy

Data for replication in this study has been deposited in OSF (https://doi.org/10.17605/OSF.IO/XWYS4).

Research involving human participants, their data, or biological material

Policy information about studies with human participants or human data. See also policy information about sex, gender (identity/presentation), and sexual orientation and race, ethnicity and racism.

Reporting on sex and gender	When conducting the behavioral experiment, we collected gender information from participants (N = 1,601). Participants identified themselves as female, male, or non-binary/unwilling to report. Gender was by no means used as a data collection criterion.<br><br>While we did not plan for a priori gender-based analysis, we have included the results of post hoc analyses in Supplementary Method 1, in compliance with the editorial policies of the Nature Portfolio (as of November 12, 2023). Our effects in the study hold for both female and male participants, while the non-binary/unwilling to report sample was too small (1.5% of the sample) to yield any statistically significant results.
Reporting on race, ethnicity, or other socially relevant groupings	This study does not incorporate race, ethnicity, or other socially relevant groups in its theorization, nor did we collect this information.
Population characteristics	Population characteristics were not used as covariates in this study. The average age of our participants was 41.9 years.
Recruitment	All participants were Amazon Mechanical Turk panelists recruited via the CloudResearch platform (formerly known as TurkPrime). We did not apply any demographic filter during the recruitment process.
Ethics oversight	Our data collection and analyses were approved by the Office of Research and Knowledge Transfer at Lingnan University, Hong Kong.

Note that full information on the approval of the study protocol must also be provided in the manuscript.

Field-specific reporting

Please select the one below that is the best fit for your research. If you are not sure, read the appropriate sections before making your selection.

☐ Life sciences ☒ Behavioural & social sciences ☐ Ecological, evolutionary & environmental sciences

For a reference copy of the document with all sections, see nature.com/documents/nr-reporting-summary-flat.pdf

Behavioural & social sciences study design

All studies must disclose on these points even when the disclosure is negative.

Study description	This study examines the impact of second-person pronoun usage in written conversations. To this end, we employ both panel data collected from papers published in Nature Communications, and data collected from participants of behavioral experiments. Both types of data are quantitative in nature.<br><br>As with the panel data, we employ difference-in-differences (DID) method to reveal that when author responses use (vs. do not use) second-person pronouns ("you"), reviewers ask fewer questions, provide briefer responses, and offer more positive and fewer negative comments. Our DID model also provides evidence that this is because "you" (vs. non-"you") usage fosters a more personal and engaging conversation.<br><br>Our preregistered behavioral experiment further demonstrates that when participants assuming the role of reviewers are addressed in second person (vs. third person), they evaluate an otherwise identical author response as more positive. This effect is mediated by the extent to which the conversation is perceived as personal and engaging.

Research sample	Our research samples reported in the main paper contain two types of data:<br><br>First, we collected revision correspondence of 13,359 papers published in Nature Communications between April 2016 (when the journal first began publishing reviewer reports) and April 2021. This dataset represents all Nature Communications papers that chose to publish their revision correspondence during said time window.<br><br>Second, we conducted a behavioral experiment on 1,601 online panelists recruited from the CloudResearch platform. Of all participants, 901 (56.3%) self-identified as female, 676 (42.2%) as male, and 24 (1.5%) as non-binary or chose not to disclose their gender; Mage = 41.9 years. This study is later replicated by another experiment reported in the Supplementary Information, with an additional 1,200 participants recruited from the same pool of panelists (of all participants, 625 [52.1%] self-identified as female, 564 [47.0%] as male, and 11 [0.9%] as non-binary; Mage = 39.9 years). No representative sampling method was employed in the two experiments.
Sampling strategy	We do not employ any sampling strategy. The peer view dataset includes all Nature Communications papers that chose to publish their revision correspondence from April 2016 through April 2021. The behavioral experiment was conducted on a sample of MTurk panelists recruited via the Cloudresearch platform, and all recorded data points were included in our analyses. No statistical method was used to predetermine sample size.
Data collection	Peer Review Data: We sourced peer review data for all papers from April 2016 to April 2021 directly from Nature Communications. Each paper's Supplementary Information section typically hosts its peer review file, which we downloaded using a custom Python (v3.7) script. These files, originally in PDF format, include both reviewer comments and author responses. To create a paper-level peer review dataset, we first separated reviewer comments from author responses for every review round and created separate TXT files for both. We then generated the variables used in our analysis for each paper by review round employing text mining techniques.<br><br>Behavioral Experiment: We recruited Amazon Mechanical Turk panelists via the CloudResearch platform, who participated in the study for monetary compensation. The survey used in the experiment was designed using Qualtrics. All participants completed the experiment from their own digital devices. All participants provided informed consent before participating in the study. The participants were unaware of their assigned condition and were not cognizant of the existence of the alternate condition to which they were not assigned. The investigators, on the other hand, were not blinded to the research hypotheses and participant allocation during experiments and outcome assessment.
Timing	We collected the peer review data in June 2021. The dataset includes all Nature Communications papers that chose to publish their revision correspondence from April 2016 through April 2021.<br><br>Data collection for the behavioral experiment in the main text (N = 1,601) was pre-registered on April 28, 2023 (Pacific Time), and was conducted from April 29 through May 1, 2023 Pacific Time.<br><br>Data collection for the behavioral experiment in the SI (N = 1,200) was conducted on July 12, 2023 Pacific Time and ended on the same day.
Data exclusions	No data is excluded from our analysis.
Non-participation	As reported by CloudResearch, the behavioral experiment in the main text (N = 1,601) has a completion rate of 90%. This means 1,779 (1,601/90%) individuals initially began the study, and 178 dropped out without completing it.<br><br>As reported by CloudResearch, the behavioral experiment in the SI (N = 1,200) has a completion rate of 92%. This means 1,304 (1,200/92%) individuals initially began the study, and 104 dropped out without completing it.
Randomization	In our behavioral experiment, participants were randomly assigned to one of two conditions, in which they were addressed by the author using either "you" or non-"you" language.

Reporting for specific materials, systems and methods

We require information from authors about some types of materials, experimental systems and methods used in many studies. Here, indicate whether each material, system or method listed is relevant to your study. If you are not sure if a list item applies to your research, read the appropriate section before selecting a response.

Materials & experimental systems

n/a	Involved in the study
☒	☐ Antibodies
☒	☐ Eukaryotic cell lines
☒	☐ Palaeontology and archaeology
☒	☐ Animals and other organisms
☒	☐ Clinical data
☒	☐ Dual use research of concern
☒	☐ Plants

Methods

n/a	Involved in the study
☒	☐ ChIP-seq
☒	☐ Flow cytometry
☒	☐ MRI-based neuroimaging
